# Stathmin expression associates with vascular and immune responses in aggressive breast cancer subgroups

**DOI:** 10.1038/s41598-020-59728-3

**Published:** 2020-02-19

**Authors:** Cecilie Askeland, Elisabeth Wik, Kenneth Finne, Even Birkeland, Jarle B. Arnes, Karin Collett, Gøril Knutsvik, Kristi Krüger, Benedicte Davidsen, Turid Aas, Geir Egil Eide, Ingunn M. Stefansson, William D. Foulkes, Lars A. Akslen

**Affiliations:** 1 Centre for Cancer Biomarkers CCBIO, Department of Clinical Medicine, Section for Pathology, University of Bergen, Bergen, N-5021 Norway; 2 Department of Pathology, Haukeland University Hospital, Bergen, N-5021 Norway; 3 Department of Surgery, Haukeland University Hospital, Bergen, N-5021 Norway; 4 Centre for Clinical Research, Haukeland University Hospital, Bergen, N-5021 Norway; 5 Department of Global Public Health and Primary Care, University of Bergen, Bergen, N-5021 Norway; 6 Department of Human Genetics, McGill University, 3640 University, Room W-315 D Montreal, Quebec, H3A 0C7 Canada

**Keywords:** Breast cancer, Cancer microenvironment, Tumour biomarkers

## Abstract

Studies indicate that stathmin expression associates with PI3K activation in breast cancer, suggesting stathmin as a marker for targetable patient subgroups. Here we assessed stathmin in relation to tumour proliferation, vascular and immune responses, *BRCA1* germline status, basal-like differentiation, clinico-pathologic features, and survival. Immunohistochemical staining was performed on breast cancers from two series (cohort 1, n = 187; cohort 2, n = 198), and mass spectrometry data from 24 cases and 12 breast cancer cell lines was examined for proteomic profiles. Open databases were also explored (TCGA, METABRIC, Oslo2 Landscape cohort, Cancer Cell Line Encyclopedia). High stathmin expression associated with tumour proliferation, p53 status, basal-like differentiation, *BRCA1* genotype, and high-grade histology. These patterns were confirmed using mRNA data. Stathmin mRNA further associated with tumour angiogenesis, immune responses and reduced survival. By logistic regression, stathmin protein independently predicted a *BRCA1* genotype (OR 10.0, p = 0.015) among ER negative tumours. Cell line analysis (Connectivity Map) implied PI3K inhibition in tumours with high stathmin. Altogether, our findings indicate that stathmin might be involved in the regulation of tumour angiogenesis and immune responses in breast cancer, in addition to tumour proliferation. Cell data point to potential effects of PI3K inhibition in tumours with high stathmin expression.

## Introduction

Breast cancer is a heterogeneous disease with different molecular subtypes^1,2^. Among these, the basal-like category represents 10–15% of all cases. Most of the basal-like tumours are triple negative (ER−/PR−/HER2−), with high histological grade and usually a more aggressive clinical behaviour. Importantly, basal-like and triple negative breast cancers are also heterogeneous^3–5^, and current studies explore novel treatment targets and improved markers for these breast cancer subtypes. Notably, there is a strong association between cases arising in heterozygotes for pathogenic *BRCA1* variants and a basal-like phenotype^6^. Henceforth, this genotype will be referred to as *BRCA1* positive.

Stathmin is a microtubule destabilizing protein important for the construction and function of the mitotic spindle^7,8^. Active, non-phosphorylated stathmin depolymerizes microtubules during interphase and late mitosis^8,9^. Tight regulation of stathmin function through phosphorylation, and de-phosphorylation is necessary for optimal function of the mitotic spindle and orderly progression through the cell cycle^9–11^. In addition to its role in mitosis, stathmin is involved in the regulation of other cellular processes such as epithelial polarity, apoptosis, and cell motility^12–14^. Further, Segatto and colleagues recently demonstrated that stathmin is required for ∆16HER2-driven early breast cancer development in mice^14^.

In breast cancer, high stathmin levels are associated with aggressive features^15–17^ and reduced survival^17–20^, and overexpression is reported in a variety of other human malignancies^12,13,21^. Regarding treatment, studies indicate that stathmin expression associates with PI3K activation, suggesting that it could be a potential marker of targetable patient subgroups. Breast cancer cell lines overexpressing stathmin exhibits decreased sensitivity to paclitaxel and vinblastine^22^, and a combination of anti-stathmin therapy and taxol has proven more effective than anti-stathmin therapy alone^23^. In a study on locally advanced breast cancer, low stathmin levels predicted response to docetaxcel-containing neoadjuvant therapy^24^.

Although stathmin plays an important role in the regulation of cell structure and function, including proliferation and hence tumour growth, little is known about its effects on the tumour microenvironment. Here, we examined stathmin expression in tumour cells as a potential marker of aggressive breast cancer subgroups. In particular, we studied the relation between stathmin and key tumour drivers such as proliferation and features of the tumour microenvironment, like angiogenesis and immune responses. Stathmin associations with basal-like phenotypes and the *BRCA1* positive genotype were explored at protein and mRNA levels. Finally, the association between stathmin and drug response patterns was mapped using cell line data, with particular focus on the PI3K pathway, and with the perspective of stathmin as a potential marker of targetable breast cancer subgroups.

## Results

### Stathmin expression in normal breast tissue

By immunohistochemistry, stathmin protein expression was mainly negative or weak in normal breast epithelial tissue. Moreover, some stathmin expression was seen in scattered immune cells and endothelial cells in benign breast tissue as well as in conjunction with carcinoma-*in-situ* changes.

### Stathmin expression is associated with aggressive tumour features

**Figure 1:**
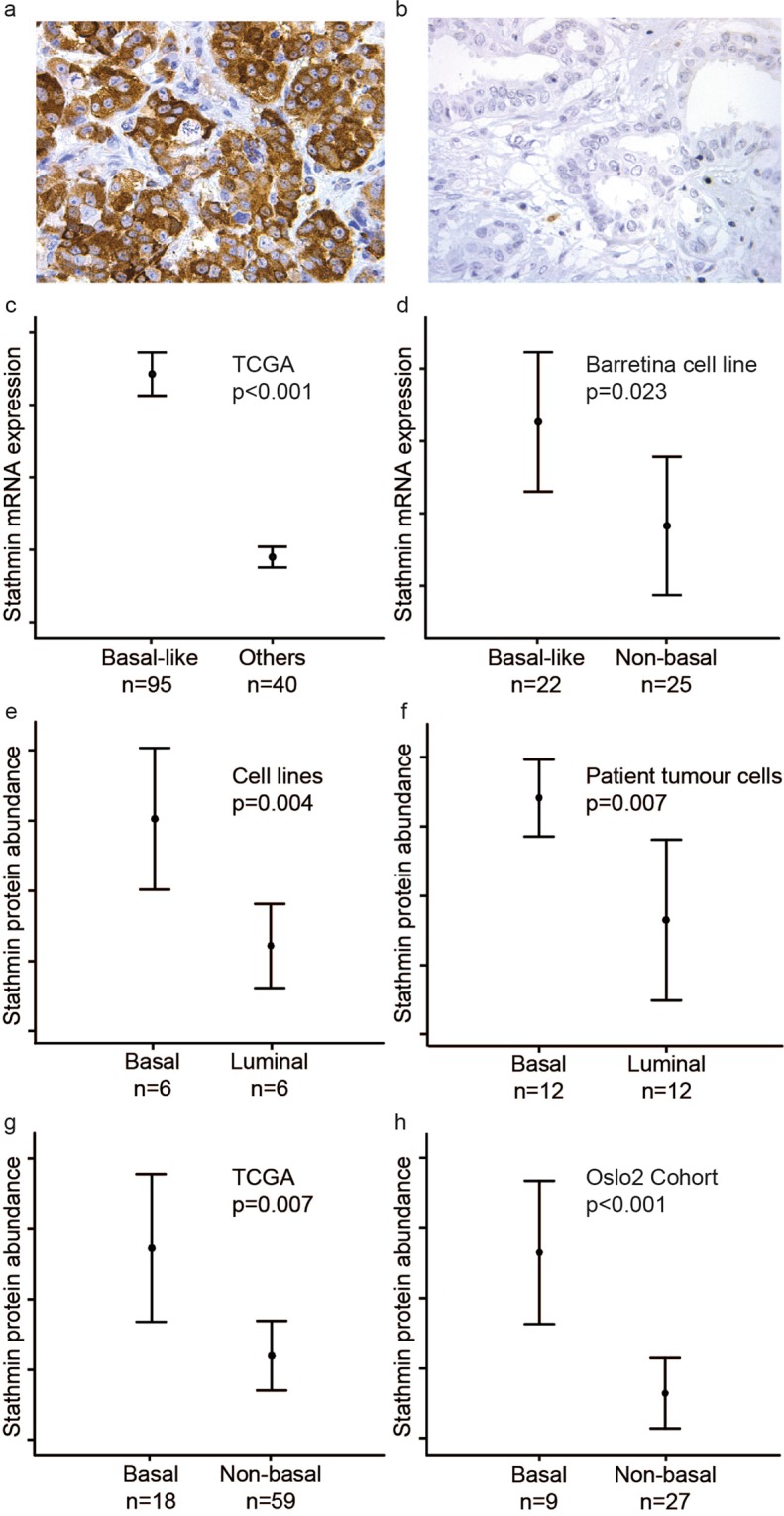
Stathmin mRNA and protein levels are significant higher in basal compared to non-basal breast cancer. Strong **(a)** and weak **(b)** cytoplasmic stain for stathmin protein in breast carcinoma. Stathmin mRNA expression in basal-like and non-basal breast cancer in the TCGA cohort **(c)** and the Barretina cell line data **(d)**. Stathmin protein abundance in basal-like compared to luminal-like cell lines **(e)**, and tumour cells from microdissected patient samples (cohort 1) **(f)**. Stathmin protein abundance in basal-like compared to non-basal tumours from patients in the CPTAC TCGA Cancer Proteome Study of Breast Tissue **(g)** and from patients in the Oslo2 Landscape cohort **(h)**. Data shown with error-bars representing 95% confidence interval of the mean, and p-values by Mann-Whitney U-test.

Immunohistochemical staining of stathmin protein was mainly localised in the tumour cell cytoplasm, and was recorded as high in 60% (112/187) and 80% (158/198) of the cases in cohort 1 and cohort 2, respectively (Fig. 1a,b**)**.

High stathmin expression was associated with high histological grade in both cohorts (p ≤ 0.002), tumour diameter > 20 mm in cohort 1 (p = 0.018), and ER (estrogen receptor) and PR (progesterone receptor) negativity in cohort 2 (p = 0.003 and p = 0.001; Table 1). In addition, high stathmin associated with strong p53 staining and a triple negative phenotype (TNP) in both cohorts (p ≤ 0.005 and p ≤ 0.012; Supplementary Tables S1–2). Stathmin was not associated with HER2 (human epidermal growth factor receptor 2) positivity, axillary lymph node status (Table 1), interval-detected tumours or locally advanced disease (data not shown).

**Table 1:** Associations between stathmin protein expression and clinico-pathological and molecular characteristics in breast cancer.

Cohort 1					
Variables	Stathmin low (n = 75)	Stathmin high (n = 112)	OR	95% CI	P-value^a^
	n (%)	n (%)			
**Tumour diameter**					0.018
≤20 mm	48 (48.0)	52 (52.0)	1		
>20 mm	27 (31.0)	60 (69.0)	2.05	1.13, 3.74	
**Hist. grade**					0.002
Grade 1–2	68 (45.6)	81 (54.4)	1		
Grade 3	7 (18.4)	31 (81.6)	3.72	1.54, 8.98	
**Nodal status**^**b**^					0.220
Neg	42 (37.2)	71 (62.8)	1		
Pos	32 (46.4)	37 (53.6)	0.68	0.37, 1.26	
**ER**					0.083
Pos (≥10%)	63 (43.4)	82 (56.6)	1		
Neg (<10%)	12 (28.6)	30 (71.4)	1.92	0.91, 4.05	
**PR**					0.554
Pos (≥10%)	52 (41.6)	73 (58.4)	1		
Neg (<10%)	23 (37.1)	39 (62.9)	1.21	0.65, 2.26	
**HER2**^**c**^					0.725
Neg	65 (40.6)	95 (59.4)	1		
Pos	10 (37.0)	17 (63.0)	1.16	0.50, 2.70	
**CK5/6**					0.006
Neg, score = 0	70 (44.3)	88 (55.4)	1		
Pos, score >0	5 (17.2)	24 (82.8)	3.82	1.39, 10.51	
**Mitotic count**^**d**^					0.001
Low, ≤5.5	63 (47.7)	69 (52.5)	1		
High, >5.5	12 (21.8)	43 (78.2)	3.27	1.58, 6.76	
**Ki67 count**^**e**^					<0.001
Low, ≤31.5%	64 (49.6)	65 (50.4)	1		
High, >31.5%	11 (19.3)	46 (80.7)	4.12	1.96, 8.66	
**Cohort 2**					
**Variables**	**Stathmin low (n** = **40)**	**Stathmin high (n** = **158)**	**OR**	**95% CI**	**P-value**^**a**^
	n (%)	n (%)			
**BRCA1 mutation**					0.001
Absent	38 (25.9)	109 (74.1)	1		
Present	2 (3.9)	49 (96.1)	8.54	1.98, 36.83	
**Tumour diameter**^**f**^					0.515
≤20 mm	24 (21.8)	86 (78.2)	1		
>20 mm	14 (17.9)	64 (82.1)	1.28	0.61, 2.66	
**Hist. grade**^**g**^					<0.001
Grade 1–2	36 (36.4)	63 (63.6)	1		
Grade 3	3 (3.5)	83 (96.5)	15.81	4.66, 53.68	
**ER**^**h**^					0.003
Pos (≥10%)	28 (28.3)	71 (71.7)	1		
Neg (<10%)	11 (11.5)	85 (88.5)	3.05	1.42, 6.55	
**PR**^**h**^					0.001
Pos (≥10%)	28 (28.9)	69 (71.1)	1		
Neg (<10%)	10 (10.3)	87 (89.7)	3.53	1.61, 7.77	
**HER2**^**i**^					0.537^j^
Neg (0–2)	38 (21.2)	141 (78.8)	1		
Pos (3)	2 (11.1)	16 (88.9)	2.16	0.48, 9.79	
**CK5/6**^**k**^					0.004
Neg, score = 0	37 (24.7)	113 (75.3)	1		
Pos, score >0	2 (4.7)	41 (95.3)	6.71	1.55, 29.11	
**Mitotic count**^**d**^					<0.001
Low (≤12.2)	38 (27.3)	101 (72.7)	1		
High (>12.2)	1 (2.2)	45 (97.8)	16.93	2.25, 127.17	

Cohort 1 (n = 187) and cohort 2 (n = 198). n: number of patients; OR: odds ratio; CI: confidence interval; ER: estrogen receptor; PR: progesterone receptor; HER2: human epidermal growth factor receptor 2; CK5/6: cytokeratin 5/6. ^a^Pearson’s chi-squared test; ^b^Five cases with missing information on nodal status; ^c^HER2 positive cases: HER2 IHC 3+ and HER2 IHC 2+ cases with a HER2/Chr17 ratio by SISH ≥ 2.0; ^d^Mitoses/mm^2^. Cut-off value by upper quartile. Thirteen cases with missing information on mitotic count in cohort 2; ^e^Cut-off value by upper quartile. One case with missing information on Ki67 count; ^f^Ten cases with missing information on tumour diameter; ^g^Thirteen cases with missing information on histological grade; ^h^Three and four cases with missing information on ER and PR status; ^i^Immunohistochemistry only. One case with missing information on HER2 status; ^j^Fisher’s exact test; ^k^Five cases with missing information on CK5/6 status.

In the Molecular Taxonomy of Breast Cancer International Consortium (METABRIC) cohorts, high stathmin mRNA expression was associated with high histological grade (both p < 0.001), tumour diameter > 20 mm (p = 0.019; validation cohort), and ER negativity both when assessing stathmin mRNA as a dichotomised variable (p < 0.001; cut-off median; Supplementary Table S3) and as a continuous variable (p ≤ 0.038). In The Cancer Genome Atlas (TCGA) cohort, stathmin mRNA expression was associated with ER negativity (p < 0.001; cut-off upper quartile; Supplementary Table S4).

### Stathmin expression is associated with a basal-like phenotype

The core basal-like phenotype (ER−, HER2−, CK5/6+ and/or EGFR+) was seen in 10% (19/184) and 20% (39/192) of the cases in cohort 1 and cohort 2, respectively. We found significant associations between high stathmin protein expression and individual basal-cell markers: CK5/6 (p ≤ 0.006; cohort 1–2) and P-cadherin (p = 0.021; cohort 2), but not with EGFR (epidermal growth factor receptor) expression (Table 1 and Supplementary Tables S1–2). High stathmin expression was significantly associated with all five immunohistochemistry-based basal-like profiles (see Supplementary Methods) in both cohorts (OR 3.71–14.28, p ≤ 0.033; Supplementary Tables S1–2).

In multiple logistic regression analysis including stathmin, mitotic count, and p53 as explanatory variables, high mitotic count (p = 0.025) and strong p53 staining (p < 0.001) were significant and independent predictors of the core basal phenotype in cohort 1, whereas stathmin (p = 0.040) was of borderline significance (Table 2). When using Ki67 as a proliferation marker and including histological grade in the model, only high Ki67 count (p = 0.003) and strong p53 staining (p = 0.002) were significant (Supplementary Table S5). In cohort 2, high stathmin expression (p = 0.001) and high p53 levels (p = 0.003) significantly and independently predicted the core basal phenotype, whereas mitotic count was not significant (Table 2).

**Table 2:** Prediction of the core basal phenotype (ER−, HER2−, CK5/6+ and/or EGFR+) by logistic regression.

Cohort 1	Unadjusted model				Adjusted model, n = 184		
Variables	n	OR	95% CI	P-value	OR	95% CI	P-value
**Mitotic count**^**a**^				<0.001			0.025
Low, ≤5.5	129	1			1		
High, >5.5	55	11.72	3.68, 37.34		4.26	1.16, 15.69	
**p53**				<0.001			<0.001
Low, score ≤3	152	1			1		
High, score >3	32	22.87	7.37, 70.95		10.70	3.14, 36.39	
**Stathmin**				<0.001			0.040
Low, score ≤4	74	1			1		
High, score >4	110	14.28	1.86, 109.51		6.41	0.75, 54.68	
**Cohort 2**	**Unadjusted model**				**Adjusted model, n** = **179**		
**Variables**	**n**	**OR**	**95% CI**	**P-value**	**OR**	**95% CI**	**P-value**
**Mitotic count**^**a**^				0.005			0.191
Low, ≤12.2	134	1			1		
High, >12.2	46	3.04	1.41, 6.57		1.74	0.76, 3.96	
**p53**				<0.001			0.003
Low, score ≤3	137	1			1		
High, score >3	54	5.61	2.65, 11.88		3.37	1.51, 7.52	
**Stathmin**				<0.001			0.001
Low, score ≤4	38	1			1		
High, score >4	154	—^b^	—		—^b^	—	

Cohort 1 (n = 187) and cohort 2 (n = 198). n: number of cases; OR: odds ratio; CI; confidence interval. ^a^Mitoses/mm^2^, cut-off value by upper quartile; ^b^Odds ratio could not be calculated due to zero BLP4 positive cases in the stathmin low group.

High stathmin mRNA expression was strongly associated with the basal-like subtype in TCGA and the two METABRIC cohorts (all p < 0.001; Fig. 1c**;** Supplementary Tables S3–4). In the breast cancer cell lines of the Cancer Cell Line Encyclopedia^25^, high stathmin mRNA associated with a basal-like phenotype (p = 0.023; Fig. 1d). Further, stathmin mRNA significantly correlated with the luminal progenitor signature score and was negatively correlated with the mature luminal signature score presented by Lim *et al*.^26^; (both p < 0.001; Supplementary Fig. S1a,b).

We found a significant association between high stathmin protein expression and positivity for the candidate stem cell marker nestin by immunohistochemistry (IHC) (both cohorts; p ≤ 0.003; Supplementary Tables S1–2). Further, stathmin mRNA correlated with a nestin signature^27^, reflecting basal-like and stemness features (p < 0.001; Supplementary Fig. S1c).

Proteomic analyses showed higher abundance of stathmin in basal-like compared to luminal-like breast cancer samples, in both in-house cell line data (n = 12; fold change = 1.8, p = 0.004; Fig. 1e) and in-house patient samples (n = 24; fold change = 4.4, p = 0.007; Fig. 1f). These results were validated by external data from the CPTAC TCGA Cancer Proteome Study of Breast Tissue^28^ and the Oslo2 Landscape cohort^29^, where both showed increased stathmin in basal-like compared to non-basal tumours (p ≤ 0.007; Fig. 1g,h). Taken together, the data indicate a consistent relationship between stathmin and a basal-like breast cancer phenotype.

From the list of genes differentially expressed between stathmin-high and -low tumours, we identified a stathmin mRNA signature composed of genes with a fold change ≤−2.0 or ≥2.0 (FDR < 0.006; 332 genes). A stathmin mRNA signature score was derived (see Supplementary Methods), and a high stathmin score was seen in basal-like tumours (p < 0.001; Supplementary Fig. S2a,b**)**. Moreover, strong correlations (positive and negative, respectively) between this stathmin score and the luminal progenitor score (p < 0.001; r(s) = 0.78), the nestin score (p < 0.001; r(s) = 0.57), and the luminal mature score (p < 0.001; r(s) = −0.84) were observed (Supplementary Fig. S2c–e). These data support a strong relation between stathmin, the basal-like phenotype, and features of stemness.

### High stathmin expression associates with germline *BRCA1* mutations

A core basal phenotype was seen in 43% (22/49) of the *BRCA1* positive cases in cohort 2. High stathmin expression associated with *BRCA1* germline mutations (OR 8.5, p = 0.001; Table 1). When adjusting for ER, mitotic count, p53 and CK5/6 in the prediction of *BRCA1* mutation status, we demonstrated a significant interaction between ER and stathmin. Notably, when stratifying the cohort by ER status, high stathmin expression alone predicted *BRCA1* mutations among ER negative tumours (p = 0.002; Table 3**)**, whereas in ER positive tumours, none of the above-mentioned markers could predict *BRCA1* positive status. Due to few *BRCA1* positive cases in the ER positive group (n = 6), we repeated the analyses using exact logistic regression to avoid small sample bias. The results were similar; in the ER negative group, only stathmin predicted *BRCA1* positive status (median unbiased estimate from exact logistic regression: OR 10.02, p = 0.015), while in the ER positive group, none of the markers were significant. Stathmin alone predicted *BRCA1* mutation status among ER negative tumours in a subgroup (n = 25) of patients under 40 years (p = 0.032). Notably, in ER negative tumours that were negative for the basal-cell marker CK5/6 (n = 53), stathmin alone predicted *BRCA1* status (p = 0.004), whereas none of the markers (stathmin, mitotic count, p53) could predict *BRCA1* status in ER negative, CK5/6 positive tumours (n = 35).

**Table 3:** Prediction of *BRCA1* germline mutation status among ER negative tumours by multiple logistic regression.

Cohort 2	Adjusted model, n = 87		
Variables	OR	95% CI	P-value
**Mitotic count**			0.543
Low, ≤12.2	1		
High, >12.2	1.33	0.53, 3.33	
**p53**			0.228
Low score, ≤3	1		
High score, >3	1.78	0.70, 4.56	
**CK5/6**			0.684
Neg, score = 0	1		
Pos, score >0	1.22	0.47, 3.13	
**Stathmin**			0.002
Low score, ≤4	1		
High score, >4	—^a^	—	

Cohort 2 (n = 198). n: number of cases; OR: odds ratio; CI: confidence interval, CK5/6: cytokeratin 5/6. ^a^Odds ratio could not be calculated due to zero stathmin low cases in the *BRCA1* positive group.

In TCGA, high stathmin mRNA associated with *BRCA1* germline mutations (OR = 3.74, p = 0.021; Supplementary Table S4). When adjusting for ER and *TP53* mutations, stathmin did not independently predict *BRCA1* mutations, possibly due to the few *BRCA1* mutated cases (n = 13) in this cohort.

### High stathmin expression associates with tumour cell proliferation

Stathmin protein levels showed strong and significant associations with tumour cell proliferation measured by mitotic count or Ki67 (p ≤ 0.001; Table 1). The median mitotic count and Ki67 levels gradually increased with increasing stathmin staining index levels (p < 0.001, both cohorts**;** Supplementary Fig. S3a–c).

**Figure 2:**
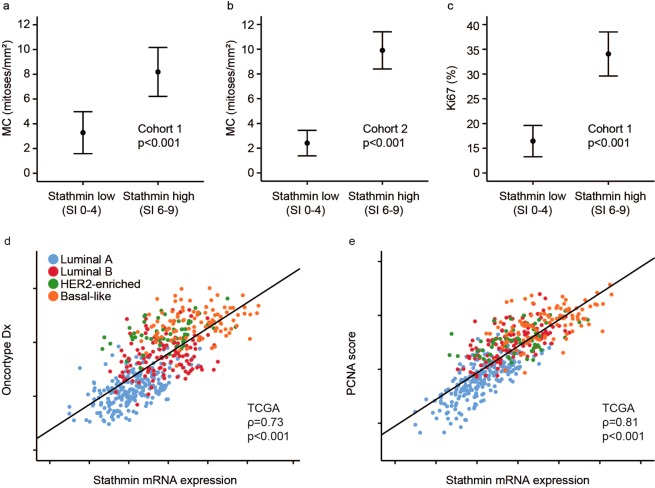
High stathmin associates with proliferation. Tumour cell proliferation by mitotic count, MC **(a,b)** and Ki67 **(c)** in stathmin low and high groups (by IHC) in breast cancers from the two patient cohorts. Correlation between stathmin mRNA expression, the Oncotype DX proliferation signature **(d)** and PCNA signature score **(e)** in the TCGA cohort. Data shown with error-bars representing 95% confidence interval of the mean, and p-values by Mann-Whitney U-test. Scatter plots are presented with p-values by Spearman’s rank correlation and the coefficients (ρ).

Mitotic count and Ki67 were significantly higher in the stathmin high compared with stathmin low group (p < 0.001; Fig. 2a–c and Supplementary Fig. S4a–c). Tumours with high stathmin had a median mitotic count of 3.8 mitoses/mm^2^ (Ki67 25%) and 6.6 mitoses/mm^2^, compared with 0.8 mitoses/mm^2^ (Ki67 13%) and 1.3 mitoses/mm^2^ in tumours with low stathmin (cohort 1 and 2, respectively). Tumours co-expressing stathmin and p53 had an especially high median mitotic count of 10.1 mitoses/mm^2^ (Ki67 60%) and 9.2 mitoses/mm^2^ compared with 2.1 mitoses/mm^2^ (Ki67 18%) and 3.3 mitoses/mm^2^ for cases without co-expression (cohort 1–2).

In multiple logistic regression analysis with stathmin, ER, p53, HER2, and CK5/6 as explanatory variables, stathmin expression (p = 0.024), HER2 status (p = 0.003) and p53 levels (p = 0.002) were significant and independent predictors of proliferation by mitotic count in cohort 1 (Table 4). Stathmin (p = 0.031), histological grade (p = 0.001), ER (p = 0.044), HER2 (p = 0.017) and p53 levels (p = 0.012) were independent predictors of proliferation by Ki67 expression (Supplementary Table S6). In cohort 2, only high stathmin (p = 0.001) and ER negativity (p < 0.001) independently predicted high proliferation by mitotic count (Table 4).

**Table 4:** Prediction of proliferation (mitotic count) by multiple logistic regression.

Cohort 1	Unadjusted model				Adjusted model, n = 187		
Variables	n	OR	95% CI	P-value	OR	95% CI	P-value
**ER**				<0.001			0.362
Pos, ≥10%	145	1			1		
Neg, <10%	42	3.73	1.82, 7.67		1.53	0.62, 3.81	
**HER2**^**a**^				0.002			0.003
Neg	160	1			1		
Pos	27	3.75	1.62, 8.68		4.25	1.66, 10.88	
**p53**				<0.001			0.002
Low, score ≤3	154	1			1		
High, score >3	33	7.33	3.23, 16.65		4.36	1.67, 11.37	
**CK5/6**				<0.001			0.287
Neg, score = 0	158	1			1		
Pos, score >0	29	3.70	2.19, 6.25		1.74	0.64, 4.78	
**Stathmin**				0.001			0.024
Low, score ≤4	75	1			1		
High, score >4	112	3.27	1.58, 6.76		2.44	1.10, 5.41	
**Cohort 2**	**Unadjusted model**				**Adjusted model, n** = **180**		
**Variables**	**n**	**OR**	**95% CI**	**P-value**	**OR**	**95% CI**	**P-value**
**ER**				<0.001			<0.001
Pos, ≥10%	95	1			1		
Neg, <10%	87	6.07	2.78, 13.26		4.72	1.92, 11.62	
**HER2**^**b**^				0.056			0.585
Neg (0–2)	166	1			1		
Pos (3)	18	2.72	1.00, 7.37		1.39	0.43, 4.47	
**p53**				0.003			0.255
Low, score ≤3	131	1			1		
High, score >3	51	3.00	1.48, 6.08		1.61	0.71, 3.63	
**CK5/6**				0.069			0.576
Neg, score = 0	140	1			1		
Pos, score >0	41	2.03	0.96, 4.30		0.77	0.30, 1.96	
**Stathmin**				<0.001			0.001
Low, score ≤4	39	1			1		
High, score >4	146	16.93	2.25, 127.17		12.72	1.62, 99.81	

Cohort 1 (n = 187) and cohort 2 (n = 198). n: number of cases; OR: odds ratio; CI: confidence interval. ^a^HER2 positive cases: HER2 IHC 3+ and HER2 IHC 2+ cases with a HER2/Chr17 ratio by SISH ≥ 2.0; ^b^Immunohistochemistry only.

When examining genes differentially expressed between stathmin-high and –low tumours, several genes involved in cell proliferation were among the top ranked upregulated genes (TCGA cohort; FDR < 0.006). In GSEA, gene sets reflecting mitosis and cell proliferation were enriched among stathmin-high cases (FDR < 0.001). Validating the GSEA output, high stathmin mRNA expression correlated with mRNA signatures reflecting tumour cell proliferation (Oncotype Dx^30^ and a PCNA score^31^; all p < 0.001; r(s) = 0.73–0.81; Fig. 2d,e).

### High expression of stathmin in tumour cells associates with vascular proliferation and immune cell activation responses

Significant positive associations between immunohistochemically detected stathmin protein in tumour cells and the two angiogenesis markers proliferative microvessel density (pMVD) (both cohorts; p ≤ 0.047) and vascular proliferation index (VPI) (cohort 2; p = 0.007) were found (Supplementary Tables S1–2).

**Figure 3:**
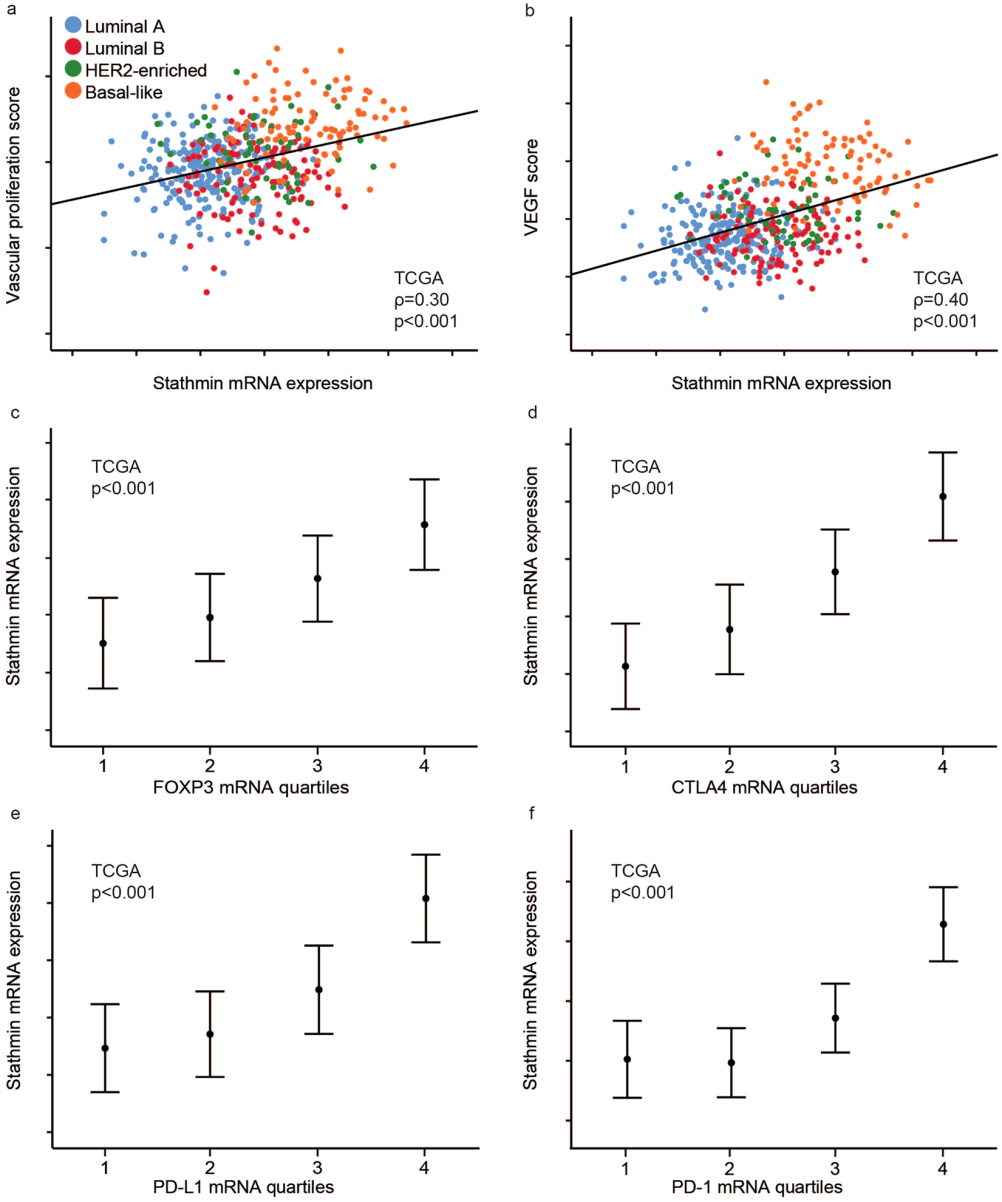
High stathmin relates to gene expression patterns reflecting vascular proliferation and immune cell activation. Correlation between stathmin mRNA expression and a gene expression vascular proliferation score **(a)** and a VEGF score **(b)**. Stathmin mRNA expression across FOXP3 **(c)**, CTLA4 **(d)**, PD-L1 **(e)** and PD-1 **(f)** mRNA quartiles. All data from the TCGA cohort. Scatter plots are presented with p-values by Spearman’s rank correlation and the coefficients (ρ). Data shown with error-bars representing 95% confidence interval of the mean, and p-values by Kruskal-Wallis test.

When exploring genes differentially enriched between stathmin-high and -low tumours, gene sets reflecting VEGF signalling and immune cell activation were enriched among stathmin-high cases (GSEA; FDR < 0.001). For validation, we demonstrated that high stathmin mRNA correlated with mRNA signatures reflecting vascular proliferation^32^ and VEGF signalling^33^ (all p < 0.001; r(s) 0.30–0.40; Fig. 3a,b), and the immune cell markers FOXP3, CTLA4, PD-L1, and PD-1 (all p < 0.001; Fig. 3c–f).

The stathmin signature correlated with stathmin mRNA expression (p < 0.001; Supplementary Fig. S2f) and strongly with two tumour cell proliferation scores (Oncotype Dx and the PCNA score; both p < 0.001; Supplementary Fig. S2g,h). Further, the stathmin signature correlated with a vascular proliferation score and a VEGF score (both p < 0.001; Supplementary Fig. S2i,j**)**. As seen for stathmin mRNA expression, high stathmin signature score was associated with higher expression of the immune cell markers FOXP3, CTLA4, PD-L1 and PD-1 (all p < 0.001; Supplementary Fig. S2k–n).

Notably, the stathmin mRNA signature score correlated stronger with tumour cell proliferation, vascular proliferation, stemness, and immune cell activation compared with stathmin mRNA expression.

Using the breast cancer cell lines of the Cancer Cell Line Encyclopedia (CCLE), the stathmin mRNA signature score was significantly associated with our vascular proliferation score, VEGF expression, hypoxia scores (Hu 2009 and Halle 2012)^34,35^, PD-L1 expression and the nestin mRNA signature score (all p < 0.001; Supplementary Fig. S5a–f). Thus, associations between the stathmin signature score and vascular markers, hypoxia scores and PD-L1 expression as well as the nestin signature score was also evident at the tumour cell level in the CCLE data, and support the results from our in-house patient cohorts and the TCGA cohort.

### High stathmin mRNA expression associates with reduced survival

Stathmin protein expression by IHC was not significantly associated with breast cancer specific survival (univariate analysis; cohort 1).

**Figure 4:**
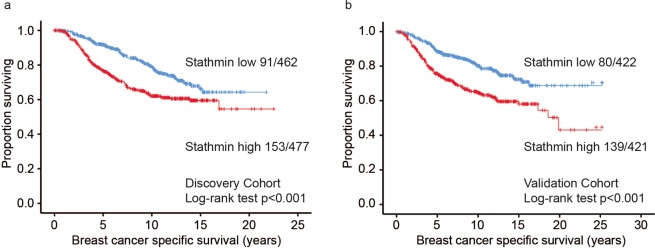
Univariate breast cancer specific survival according to stathmin mRNA status. Kaplan-Meier univariate breast cancer specific survival analysis in the METABRIC discovery **(a)** and validation cohorts **(b)** according to stathmin mRNA expression (cut-point by median, log-rank test for difference). For each category, the number of breast cancer deaths is given, followed by the total number of cases in each category.

In METABRIC cohorts, high stathmin mRNA associated with shorter disease specific survival, both when assessed as a continuous variable (both cohorts: HR 1.5; 95% CI 1.3–1.8, p < 0.001) and when dichotomised (discovery cohort: HR 1.4; 95% CI 1.4–2.4, p < 0.001; validation cohort: HR 2.0; 95% CI 1.5–2.7, p < 0.001; Fig. 4). Stathmin mRNA expression also predicted outcome in the datasets of the “KM plotter” database^36^ (Supplementary Fig. S6). In multivariate survival analyses (METABRIC cohorts), adjusting for tumour size, histological grade and lymph node status, high stathmin mRNA expression independently associated with shorter disease specific survival (discovery cohort: HR = 1.6, 95% CI 1.2–2.1; p = 0.001; validation cohort: HR = 1.7, 95% CI 1.3–2.3; p < 0.001). In addition, when also adjusting for molecular subtypes, stathmin maintained its prognostic information with borderline significance in the discovery cohort (HR = 1.4, 95% CI 0.99–1.3; p = 0.057).

### Compounds with potential PI3K inhibitory properties in tumours with high stathmin

We queried the drug signature database Connectivity Map^37^ (version 02) for drug-effect gene expression profiles anti-correlated to genes differentially expressed between high and low stathmin mRNA expression. Among 1309 small molecules represented in Connectivity Map, expression profiles from compounds with PI3K inhibitory properties were top ranked (TCGA; Supplementary Table 7). These compounds might have a potential to drive stathmin-high tumours into a stathmin-low state, with less aggressive phenotype, proposing a potential relevance for PI3K inhibitors in stathmin-high tumours.

## Discussion

Stathmin is a known regulator of the mitotic spindle and hence cellular proliferation. In relation to breast cancer, stathmin has been associated with aggressive features such as large tumour size^15^, high histological grade^15,16^, hormone receptor negativity^15–17,20^, basal-like and triple negative phenotypes^17^, as well as reduced survival in some studies^18–20,38^. Here, we show for the first time that stathmin protein expression by IHC is strongly related to *BRCA1* positive breast cancers. The finding is further supported by our data that stathmin mRNA is associated with *BRCA1* positive status in the TCGA-cohort, in line with one small mRNA-based study by Bane *et al*.^39^. In a multivariate prediction model, we show that stathmin protein assessed by IHC is independently associated with *BRCA1* status among ER negative tumours. In this model, the basal-like phenotype, by CK5/6 expression, was not significant. Larger studies are needed to elucidate the predictive value of stathmin among ER positive cases.

It has been suggested that stathmin expression segregates with features of less differentiated tumours with stem cell characteristics^17^. This is supported by our results, showing significant associations between stathmin and the stemness marker nestin, which is reported to be strongly associated with *BRCA1* positive status, basal-like phenotypes, and reduced survival^27^. We also found strong associations between stathmin and basal-like breast cancer profiles present in two in-house patient cohorts, in proteomics data from our own as well as external cohorts, from cell line studies, and in public mRNA data.

As predicted, our results point to strong associations between stathmin protein and markers of tumour cell proliferation in breast cancer such as Ki67 expression and mitotic count. Notably, stathmin protein showed an independent value among other markers in prediction of tumour proliferation. Similar findings were found at the mRNA level, where associations with proliferation related gene signatures such as Oncotype Dx and the PCNA score were seen. Results from a study by Miceli and colleagues supports that stathmin leads to increased proliferation *in vitro* and tumour growth *in vivo*. First, they showed that adenovirus-mediated delivery of an anti-stathmin ribozyme gene into breast cancer cell lines decreased proliferation irrespective of the ER status. Next, they demonstrated reduced growth of mammary tumours in nude mice after anti-stathmin therapy^23^. In a clinical study on lapatinib, a decrease in stathmin level was associated with Ki67 reduction in HER2 negative breast cancer. Pre-treatment levels of stathmin were, however, not predictive of response to lapatinib treatment^40^.

She and colleagues recently reported that stathmin protein expression was significantly higher in tumours with PTEN protein loss, as well as in tumours expressing a PTEN loss gene expression signature^41^. Stathmin has previously been suggested as a protein marker for PTEN loss and is therefore suggested as a useful test for PI3K pathway activation^18^. PI3K inhibitors as monotherapy in breast cancer, including in triple negative cancer, have so far not demonstrated significant efficacy in clinical trials^42,43^, although combinations of PI3K inhibitors with other targeted drugs have shown promising results, such as the combination between PI3K inhibitors and fulvestrant^43,44^. A combination of PI3K/mTOR- and PARP inhibitors has been suggested to improve outcomes in breast cancer patients with *BRCA* germline mutations, a basal-like or a triple negative phenotype^45–47^. Notably, we found that compounds with PI3K inhibitory properties negatively correlated with the transcriptional pattern of stathmin-high cases (Connectivity Map analyses), suggesting adding PI3K inhibitors as a treatment strategy with potential relevance in stathmin-high tumours.

Some^18–20,38^, but not all^16^, previous studies have shown a correlation between stathmin expression and prognosis in breast cancer. Here, in METABRIC cohorts, high stathmin mRNA was associated with shorter disease specific survival, also when adjusted for tumour size, histological grade and lymph node status. In contrast, we found no significant association between high stathmin protein expression and reduced breast cancer specific survival in our in-house series (cohort 1), perhaps due to limited sample size. A recent meta-analysis addressed the prognostic role of stathmin in patients with solid cancers, including breast cancer^48^. In tumour-type subgroup analysis, stathmin was associated with worse disease-free survival in breast cancer. As there were few eligible breast cancer studies for inclusion, the authors could not perform tumour-type subgroup analysis for overall survival or disease-specific survival. Thus, there is a need for large breast cancer studies to determine the prognostic role of stathmin.

Previous studies have focused mainly on the relationship between stathmin and tumour cell proliferation. Here, we show that stathmin expression in tumour cells might also be important for and influence processes in the tumour microenvironment, such as angiogenesis and immune responses, which to our knowledge is not well documented in breast cancer. We found that stathmin was significantly associated with VEGF and vascular proliferation as well as with the immune response markers FOXP3, CTLA4, PD-L1 and PD-1 in tumour tissues. Notably, the stathmin mRNA signature was also associated with vascular markers, hypoxia scores, and PD-L1 expression in breast cancer cell lines. These findings might in part reflect the relationship between stathmin and ER negative tumours with basal-like and stemness features, since these are known for increased immune cell content and elevated angiogenesis^49–52^. Such complex interactions might be relevant for treatment possibilities and potential drug combination strategies.

More direct mechanistic relationships should be further explored between stathmin and tumour microenvironment properties. Previously, stathmin was found to control the regulation of hypoxia inducible factor (HIF)-1α through the PI3K/Akt/mTOR pathway in ovarian cancer cells^53^, and stathmin knockdown supressed expression of HIF-1α and vascular endothelial growth factor (VEGF) and impeded phosphorylation of ribosomal protein S6 kinase 1 (S6K) and Akt. Stathmin overexpression, however, upregulated expression of HIF-1α and VEGF^53^. In relation to immune responses, stathmin expression was correlated to PD-L1 levels in a study of head and neck squamous cell carcinoma, suggesting that there might be a relationship between stathmin and the immune system, and that stathmin might be associated with immune suppression^54^.

In conclusion, our study shows that stathmin expression is associated with several high-grade features of breast cancer, such as increased proliferation, ER negativity, and basal-like phenotypes. A strong and novel relationship between stathmin protein and *BRCA1* positive ER negative cancers was found. Links between transcriptional patterns of high stathmin and cell line exposure data from The Connectivity Map implies that PI3K inhibition might be of relevance in tumours with high stathmin expression. Finally, stathmin might also be involved in the regulation of tumour angiogenesis and immune responses, as important co-factors in breast cancer progression.

## Methods

### Patient cohorts

The study includes 385 primary breast cancers from two independent cohorts. Cohort 1 (n = 187), which has been previously described by our group^51,55–57^, is a nested case-control study based on the Norwegian Mammography Screening Program in Hordaland County during 1996–2001. Briefly, 95 invasive interval cancers were matched by tumour diameter (±2.0 mm) with 95 screen-detected cancers from a total of 317 invasive tumours that occurred during the first two screening rounds. Three of the 190 cases were excluded due to different reasons. Outcome data were included from the Norwegian Cause of Death Registry (survival time, status, cause of death). Last date of follow-up was June 30, 2017 (median follow-up time of survivors, 222 months; range 191–255). During follow-up, 46 patients (25%) died of breast cancer, and 40 (21%) died of other causes. Cohort 2 (n = 198), which has been described previously^27^, is a case-control study that initially included 53 *BRCA1* positive breast cancers, 45 *BRCA2* positive and 53 *BRCA* mutation negative cases; primary breast cancers were collected from patients counselled at the Hereditary Cancer Clinic at the McGill University Health Centre (MUHC) and the Jewish General Hospital, Montreal, Canada during 1986–2005. In addition, 51 *BRCA* mutation negative primary breast cancers from our own archive were included. The three groups were balanced with regards to age and size, but a strict case-by-case matching procedure could not be accomplished due to a small pool of available controls. Four cases lacked sufficient tumour tissue for stathmin staining and were excluded from the analyses. Follow-up information was not available for this cohort. The study was approved by the Institutional Review Board at McGill University Hospital, A03-M33-02A, and the Western Regional Committee for Medical and Health Research Ethics, REC West (REK 2014/1984). The informed consent was waived by the REK ethics committee. All studies were performed in accordance with guidelines and regulations by the University of Bergen and REK, and in accordance with the Declaration of Helsinki Principles.

### Immunohistochemistry

#### Immunohistochemical staining for stathmin protein

In both series, stathmin staining was performed manually on five μm thin tissue microarray (TMA) sections from formalin-fixed and paraffin-embedded tumour tissue^58^. Whole sections were used in cases with poor quality or insufficient tumour material for evaluation in the TMA cores (26 cases in cohort 1, and 18 cases in cohort 2). The sections were deparaffinised in xylene, rehydrated through a series of graded alcohols and rinsed in distilled water. Microwave oven heating with Tris-EDTA, pH 9.0, at 750 W for 10 minutes followed by 350 W for 15 minutes was used for epitope retrieval. To reduce background staining, a peroxidase-blocking agent (DM801) and a protein block (X0909) were applied before the primary antibody. The TMA sections were incubated at room temperature for 60 minutes using a polyclonal rabbit antibody against stathmin (#3352) from Cell Signaling Technology, diluted 1:50. After rinsing with a wash buffer solution, EV FLEX+ Rabbit LINK (DM 805) was added for 30 minutes followed by a new rinsing before the EV FLEX/HPR (DM802) was used for 30 min. To add colour at the site of the target antigen recognised by the primary antibody, AEC (Chromogen) K4009 was applied. Finally, sections were rinsed in distilled water and counterstained with Haematoxylin (S2020).

#### Evaluation of staining

Stathmin staining was recorded using a semi-quantitative and subjective grading system, considering the intensity of staining (none = 0, weak = 1, moderate = 2 and strong = 3) and the proportion of tumour cells showing a positive reaction (<10% = 1, 10–50% = 2, >50% = 3). A staining index (values 0–9) was obtained as a product of staining intensity (0–3) and proportion of immunopositive cells (1–3). Stathmin expression was considered positive (high) for staining index ≥6 (median in cohort 1, and lower quartile in cohort 2) based on frequency distributions. Information on other histological variables is presented in Supplementary Methods.

### Proteomics studies of laser-capture microdissected paraffin embedded tumour tissue

Twenty-four formalin-fixed, paraffin-embedded (FFPE) breast cancer specimens were selected (twelve basal-like, six luminal A and six luminal B). All basal-like samples were also triple negative, and all luminal samples were ER and PR positive, and HER2 negative. The luminal B tumours displayed more than 15% Ki67-positive nuclei (whole section). All were diagnosed as invasive carcinomas (NST; previously ductal carcinomas). Ten μm thick FFPE sections were deparaffinised, rehydrated and stained with haematoxylin. Breast cancer epithelium was laser micro-dissected (PALM MicroBeam, Zeiss) and pressure catapulted into a tube cap (AdhesiveCap 500 opaque, Zeiss). Depending on the available tissue, 0.8–1.9 × 10^7^ µm^3^ were micro-dissected.

### Proteomics studies of breast cancer cell lines

Basal-like breast cancer cell lines (MDA-MB-231, MB-468, BT-549, SUM-1315, SUM-159 and Hs 578 T) and luminal like breast cancer cell lines (MCF7, T47D, HCC1428, SK-Br-3, ZR-73-50 and BT-474) were obtained from the American Type Culture Collection (ATCC, Manassas, VA). Information on how the cell lines were cultured, and methods used for protein extraction, digestion and mass spectrometry analysis is presented in Supplementary Methods.

### Proteomic data analyses

Raw data from the mass spectrometer were processed using the MaxQuant software (v1.6.0.16) with recommended settings for label-free quantification^59^. Identified features were searched against an up-to-date human “reference proteome”-database from UniProt.org. Hands-on statistical analyses of processed mass spectrometry data were performed using the Perseus software (v1.6.0.7)^60^.

#### External proteomic datasets

Data from the CPTAC TCGA Cancer Proteome Study of Breast Tissue and the Oslo2 Landscape cohort were used for validation. The CPTAC TCGA Cancer Proteome Study of Breast Tissue dataset was downloaded from Clinical Proteomic Tumour Analysis Consortium (NCI/NIH), and consists of 105 breast cancer samples, which were reduced to 77 after quality control by Mertins *et al*.^28^ 18 basal-like samples, 12 HER2 enriched, 23 luminal A and 24 luminal B. Data from the Oslo2 Landscape cohort was downloaded from www.breastcancerlandscape.org/. It includes 45 breast cancer samples: nine basal-like, nine HER2 enriched, nine luminal A, nine luminal B and nine normal breast-like samples^29^. The normal breast-like samples were excluded from analyses.

### Gene expression resources

For the exploration of transcriptional patterns related to stathmin in breast cancer, publicly available mRNA gene expression datasets with information on clinico-pathologic and follow-up data and molecular subtypes were analysed. (1) The TCGA cohort (n = 505)^61^; (2) the METABRIC cohorts (discovery cohort, n = 939; validation cohort, n = 845)^62^. Intrinsic molecular subtypes based on PAM50 classification were available^63^. The normal breast-like category was excluded. Transcriptomic data from the breast cancer cell lines in the Cancer Cell Line Encyclopedia (n = 47 with information on molecular subtypes)^25^ was also explored. An online database, “KM plotter” (www.kmplot.com)^36^, including EGA (European Genome-phenome Archive) and GEO datasets (Affymetrix microarrays only), was used to evaluate stathmin mRNA in relation to recurrence-free breast cancer survival in a merged dataset of 3951 breast cancer cases. Cut-off point for analysis with dichotomised stathmin mRNA was defined after considering frequency distributions and survival pattern of quartiles (TCGA; upper quartile, METABRIC; median).

Differentially expressed genes between cases with high versus low stathmin mRNA expression were identified based on Significance Analysis of Microarrays (SAM)^64^. Gene sets significantly enriched in cases with high stathmin mRNA expression were explored by applying the Gene Set Enrichment Analysis (GSEA; www.broadinstitute.org/gsea)^65^ and the signatures of Molecular Signatures Database (MSigDB; www.broadinstitute.org/gsea/msigdb). Multiple probes covering the same gene were collated accordingly max probe^65^.

### Gene expression data analyses

Details on methods applied in gene expression analysis are presented in Supplementary Methods.

### Statistical methods

Data were analysed using the SPSS Statistics for Windows, Version 25.0. (IBM Corp., Armonk, NY, USA). Statistical significance was assessed at the two-sided 5% level, whereas borderline statistical significance was defined as P-values between 5% and 10%. Associations between categorical variables were evaluated using the Pearson’s χ² test or Fisher’s exact test, as appropriate. Non-parametric correlations were tested by the Spearman’s rank coefficient. Continuous variables with a non-normal distribution were compared between two groups using the Mann-Whitney U test, and between more than two groups using Kruskal-Wallis H test. Multiple logistic regression analysis was utilised for prediction of BRCA1 germline mutation status, the basal-like phenotype and tumour cell proliferation. The calculations were done according to the Backward Elimination (Likelihood Ratio) method, with p-values derived from Step1 in the “model if term removed”-table. Tests for interactions between stathmin and selected co-variables were performed. Prediction of *BRCA1* positive status was also performed using exact logistic regression in Stata 16 due to few *BRCA1* positive cases in the ER positive subgroup. The end-point in survival analyses was breast cancer specific survival (BCSS) in patient cohort 1 and METABRIC, and recurrence-free survival (RFS) in “KM plotter”. One patient with distant metastases at time of diagnosis was excluded from analysis in patient cohort 1. Univariate survival analyses were carried out using the Kaplan-Meier method with significance determined by the log-rank test. Entry date was the date of diagnosis. Patients who died from other causes were censored at the date of death. Multivariate survival analyses were performed on both continuous and dichotomised stathmin mRNA-data from METABRIC using Cox’ proportional hazards regression model. Multivariate analyses adjusted for molecular subtypes in addition to tumour diameter, histological grade and lymph node status. Only patients with information on all variables were included in the analyses.

## Supplementary information

Supplementary Information.

## References

[1] Perou CM (2000). Molecular portraits of human breast tumours. Nat..

[2] Sorlie T (2001). Gene expression patterns of breast carcinomas distinguish tumor subclasses with clinical implications. Proc. Natl Acad. Sci. U S Am..

[3] Lehmann BD (2011). Identification of human triple-negative breast cancer subtypes and preclinical models for selection of targeted therapies. J. Clin. Invest..

[4] Lehmann BD (2016). Refinement of Triple-Negative Breast Cancer Molecular Subtypes: Implications for Neoadjuvant Chemotherapy Selection. PLoS one.

[5] Burstein MD (2015). Comprehensive genomic analysis identifies novel subtypes and targets of triple-negative breast cancer. Clin. Cancer Res..

[6] Foulkes WD (2003). Germline BRCA1 mutations and a basal epithelial phenotype in breast cancer. J. Natl Cancer Inst..

[7] Belmont LD, Mitchison TJ (1996). Identification of a protein that interacts with tubulin dimers and increases the catastrophe rate of microtubules. Cell.

[8] Marklund U, Larsson N, Gradin HM, Brattsand G, Gullberg M (1996). Oncoprotein 18 is a phosphorylation-responsive regulator of microtubule dynamics. EMBO J..

[9] Mistry SJ, Atweh GF (2001). Stathmin inhibition enhances okadaic acid-induced mitotic arrest: a potential role for stathmin in mitotic exit. J. Biol. Chem..

[10] Mistry SJ, Atweh GF (2002). Role of stathmin in the regulation of the mitotic spindle: potential applications in cancer therapy. Mt. Sinai J. Med..

[11] Rubin CI, Atweh GF (2004). The role of stathmin in the regulation of the cell cycle. J. Cell Biochem..

[12] Belletti B, Baldassarre G (2011). Stathmin: a protein with many tasks. New biomarker and potential target in cancer. Expert. Opin. therapeutic targets.

[13] Biaoxue R, Xiguang C, Hua L, Shuanying Y (2016). Stathmin-dependent molecular targeting therapy for malignant tumor: the latest 5 years’ discoveries and developments. J. Transl. Med..

[14] Segatto I (2019). Stathmin Is Required for Normal Mouse Mammary Gland Development and Delta16HER2-Driven Tumorigenesis. Cancer Res..

[15] Brattsand G (2000). Correlation of oncoprotein 18/stathmin expression in human breast cancer with established prognostic factors. Br. J. cancer.

[16] Curmi PA (2000). Overexpression of stathmin in breast carcinomas points out to highly proliferative tumours. Br. J. cancer.

[17] Obayashi S (2017). Stathmin1 expression is associated with aggressive phenotypes and cancer stem cell marker expression in breast cancer patients. Int. J. Oncol..

[18] Saal LH (2007). Poor prognosis in carcinoma is associated with a gene expression signature of aberrant PTEN tumor suppressor pathway activity. Proc. Natl Acad. Sci. U S Am..

[19] Golouh R (2008). The prognostic value of Stathmin-1, S100A2, and SYK proteins in ER-positive primary breast cancer patients treated with adjuvant tamoxifen monotherapy: an immunohistochemical study. Breast cancer Res. Treat..

[20] Baquero MT (2012). Stathmin expression and its relationship to microtubule-associated protein tau and outcome in breast cancer. Cancer.

[21] Rana S, Maples PB, Senzer N, Nemunaitis J (2008). Stathmin 1: a novel therapeutic target for anticancer activity. Expert. Rev. Anticancer. Ther..

[22] Alli E, Bash-Babula J, Yang JM, Hait WN (2002). Effect of stathmin on the sensitivity to antimicrotubule drugs in human breast cancer. Cancer Res..

[23] Miceli C, Tejada A, Castaneda A, Mistry SJ (2013). Cell cycle inhibition therapy that targets stathmin in *in vitro* and *in vivo* models of breast cancer. Cancer gene Ther..

[24] Meng XL (2012). Low expression of stathmin in tumor predicts high response to neoadjuvant chemotherapy with docetaxel-containing regimens in locally advanced breast cancer. Genet. Test. Mol. Biomarkers.

[25] Barretina J (2012). The Cancer Cell Line Encyclopedia enables predictive modelling of anticancer drug sensitivity. Nat..

[26] Lim E (2009). Aberrant luminal progenitors as the candidate target population for basal tumor development in BRCA1 mutation carriers. Nat. Med..

[27] Kruger K (2017). Expression of Nestin associates with BRCA1 mutations, a basal-like phenotype and aggressive breast cancer. Sci. Rep..

[28] Mertins P (2016). Proteogenomics connects somatic mutations to signalling in breast cancer. Nat..

[29] Johansson HJ (2019). Breast cancer quantitative proteome and proteogenomic landscape. Nat. Commun..

[30] Paik S (2004). A multigene assay to predict recurrence of tamoxifen-treated, node-negative breast cancer. N. Engl. J. Med..

[31] Venet D, Dumont JE, Detours V (2011). Most random gene expression signatures are significantly associated with breast cancer outcome. PLoS Comput. Biol..

[32] Stefansson IM (2015). Increased angiogenesis is associated with a 32-gene expression signature and 6p21 amplification in aggressive endometrial cancer. Oncotarget.

[33] Hu Z (2009). A compact VEGF signature associated with distant metastases and poor outcomes. BMC Med..

[34] Eustace A (2013). A 26-gene hypoxia signature predicts benefit from hypoxia-modifying therapy in laryngeal cancer but not bladder cancer. Clin. Cancer Res..

[35] Halle C (2012). Hypoxia-induced gene expression in chemoradioresistant cervical cancer revealed by dynamic contrast-enhanced MRI. Cancer Res..

[36] Gyorffy B (2010). An online survival analysis tool to rapidly assess the effect of 22,277 genes on breast cancer prognosis using microarray data of 1,809 patients. Breast cancer Res. Treat..

[37] Lamb J (2006). The Connectivity Map: using gene-expression signatures to connect small molecules, genes, and disease. Sci..

[38] Kuang XY (2015). Stathmin and phospho-stathmin protein signature is associated with survival outcomes of breast cancer patients. Oncotarget.

[39] Bane AL (2009). Expression profiling of familial breast cancers demonstrates higher expression of FGFR2 in BRCA2-associated tumors. Breast cancer Res. Treat..

[40] Leary A (2015). Antiproliferative Effect of Lapatinib in HER2-Positive and HER2-Negative/HER3-High Breast Cancer: Results of the Presurgical Randomized MAPLE Trial (CRUK E/06/039). Clin. Cancer Res..

[41] She QB (2016). Integrated molecular pathway analysis informs a synergistic combination therapy targeting PTEN/PI3K and EGFR pathways for basal-like breast cancer. BMC cancer.

[42] Costa RLB, Han HS, Gradishar WJ (2018). Targeting the PI3K/AKT/mTOR pathway in triple-negative breast cancer: a review. Breast cancer Res. Treat..

[43] Qin H (2018). The impact of PI3K inhibitors on breast cancer cell and its tumor microenvironment. PeerJ.

[44] Andre F (2019). Alpelisib for PIK3CA-Mutated, Hormone Receptor-Positive Advanced Breast Cancer. N. Engl. J. Med..

[45] Ibrahim YH (2012). PI3K inhibition impairs BRCA1/2 expression and sensitizes BRCA-proficient triple-negative breast cancer to PARP inhibition. Cancer discovery.

[46] Juvekar A (2012). Combining a PI3K inhibitor with a PARP inhibitor provides an effective therapy for BRCA1-related breast cancer. Cancer discovery.

[47] Matulonis UA (2017). Phase I dose escalation study of the PI3kinase pathway inhibitor BKM120 and the oral poly (ADP ribose) polymerase (PARP) inhibitor olaparib for the treatment of high-grade serous ovarian and breast cancer. Ann. oncology: Off. J. Eur. Soc. Med. Oncol..

[48] Mao Q (2018). Prognostic Role of High Stathmin 1 Expression in Patients with Solid Tumors: Evidence from a Meta-Analysis. Cell Physiol. Biochem..

[49] Miyan M, Schmidt-Mende J, Kiessling R, Poschke I, de Boniface J (2016). Differential tumor infiltration by T-cells characterizes intrinsic molecular subtypes in breast cancer. J. Transl. Med..

[50] Nalwoga H (2011). Vascular proliferation is increased in basal-like breast cancer. Breast cancer Res. Treat..

[51] Kruger K (2013). Microvessel proliferation by co-expression of endothelial nestin and Ki-67 is associated with a basal-like phenotype and aggressive features in breast cancer. Breast.

[52] Bujor IS (2018). Evaluation of Vascular Proliferation in Molecular Subtypes of Breast Cancer. Vivo.

[53] Tamura K, Yoshie M, Miyajima E, Kano M, Tachikawa E (2013). Stathmin Regulates Hypoxia-Inducible Factor-1alpha Expression through the Mammalian Target of Rapamycin Pathway in Ovarian Clear Cell Adenocarcinoma. ISRN Pharmacol..

[54] Wu H, Deng WW, Yang LL, Zhang WF, Sun ZJ (2018). Expression and phosphorylation of Stathmin 1 indicate poor survival in head and neck squamous cell carcinoma and associate with immune suppression. Biomark Med..

[55] Collett K (2005). A basal epithelial phenotype is more frequent in interval breast cancers compared with screen detected tumors. Cancer Epidemiol. Biomarkers Prev..

[56] Collett K (2006). Expression of enhancer of zeste homologue 2 is significantly associated with increased tumor cell proliferation and is a marker of aggressive breast cancer. Clin. Cancer Res..

[57] Arnes JB, Collett K, Akslen LA (2008). Independent prognostic value of the basal-like phenotype of breast cancer and associations with EGFR and candidate stem cell marker BMI-1. Histopathology.

[58] Camp RL, Neumeister V, Rimm DL (2008). A decade of tissue microarrays: progress in the discovery and validation of cancer biomarkers. J. Clin. oncology: Off. J. Am. Soc. Clin. Oncol..

[59] Cox J (2014). Accurate proteome-wide label-free quantification by delayed normalization and maximal peptide ratio extraction, termed MaxLFQ. Mol. Cell. proteomics: MCP.

[60] Tyanova S (2016). The Perseus computational platform for comprehensive analysis of (prote)omics data. Nat. Methods.

[61] Comprehensive molecular portraits of human breast tumours. *Nature* **490**, 61-70 (2012). 10.1038/nature11412 PMC3465532 23000897

[62] Curtis C (2012). The genomic and transcriptomic architecture of 2,000 breast tumours reveals novel subgroups. Nat..

[63] Parker JS (2009). Supervised risk predictor of breast cancer based on intrinsic subtypes. J. Clin. oncology: Off. J. Am. Soc. Clin. Oncol..

[64] Tusher VG, Tibshirani R, Chu G (2001). Significance analysis of microarrays applied to the ionizing radiation response. Proc. Natl Acad. Sci. United States of America.

[65] Subramanian A (2005). Gene set enrichment analysis: a knowledge-based approach for interpreting genome-wide expression profiles. Proc. Natl Acad. Sci. United States of America.

