# Supplementary material for: Stathmin expression associates with vascular and immune responses in aggressive breast cancer subgroups
Source: Sci Rep. 2020 Feb 19;10:2914. doi: 10.1038/s41598-020-59728-3 (PMC7031232; doi:10.1038/s41598-020-59728-3)
Supplement: Supplementary file 1 — Supplementary Information. [file 41598_2020_59728_MOESM1_ESM.pdf]

Supplementary Information

Stathmin expression associates with vascular and immune responses in aggressive breast cancer subgroups

Cecilie Askeland[1,2], Elisabeth Wik[1,2], Kenneth Finne[1], Even Birkeland[1], Jarle B. Arnes[2], Karin Collett[2], Gøril Knutsvik[2], Kristi Krüger[1], Benedicte Davidsen[3], Turid Aas[3], Geir Egil Eide[4,5], Ingunn M. Stefansson[1,2], William D. Foulkes[6], Lars A. Akslen*[,1,2]

[1] Centre for Cancer Biomarkers CCBIO, Department of Clinical Medicine, Section for Pathology, University of Bergen, Bergen N-5021, Norway

[2] Department of Pathology, Haukeland University Hospital, Bergen N-5021, Norway

[3] Department of Surgery, Haukeland University Hospital, Bergen N-5021, Norway

[4] Centre for Clinical Research, Haukeland University Hospital, Bergen N-5021, Norway

[5] Department of Global Public Health and Primary Care, University of Bergen, Bergen N-5021, Norway

[6] Department of Human Genetics, McGill University, 3640 University, Room W-315 D Montreal, Quebec H3A 0C7, Canada

Supplementary Methods

Immunohistochemistry

Histologic variables. In both cohorts, data on several tumour markers were available from previous studies: staining for human epidermal growth factor receptor 2 (HER2), p53, cytokeratin 5/6 (CK5/6), P-cadherin, epidermal growth factor receptor (EGFR) and nestin were performed on TMA slides. Dual stained whole sections (nestin and Ki67 in cohort 1, and factor VIII and Ki67 in cohort 2) were used to evaluate proliferative microvessel density (pMVD) and vascular proliferation index (VPI=pMVD/MVD). Mitotic count (reported as mitoses per mm^2) from H&E-stained whole sections were used as measure for tumour cell proliferation. For cohort 1, estrogen receptor (ER) and progesterone receptor (PR) data were included from the routine pathology reports. Ki67 staining was done on whole sections. In cohort 2, ER and PR were stained on TMA slides. The antibodies applied, and staining and evaluation methods used for biomarker assessment in cohorts 1 and 2 are presented in previous publications[1-11]. For p53, nestin, pMVD, VPI, CK5/6, P-cadherin, HER2, EGFR, mitotic count and Ki67 we used cut-points as previously described[3,5,9-12]. The cut-point for ER and PR positivity was kept at 10 % in this research study, despite the ASCO/CAP guidelines[13], since studies have reported that tumours with ER < 1% have characteristics similar to those with ER 1-10%[14,15].

Inter-observer agreement for stathmin immunostaining. For stathmin, a subset of both cohorts (n=87 and n=42) was scored independently by two observers (C.A. and K.C.). The inter-observer agreement (kappa coefficient) between negative (low SI) and positive (high SI) cases was 0.75 for cohort 1 (46% of cases scored by both observers)

and 0.78 for cohort 2 (21% of cases scored by both observers). In cases recorded with different values, each case was discussed and a consensus was achieved.

Basal-like phenotypes. Five previously described immunohistochemistry-based basal-like profiles (**BLP 1-5**) were used as surrogate markers of the intrinsic basal-like subgroup defined by gene expression patterns[3,10]. All five basal-like profiles were negative for ER and HER2. Additionally, **BLP 1** showed positivity for CK5/6, **BLP 2** for P-cadherin and **BLP 3** for EGFR. **BLP 4,** also defined as the core basal phenotype (CBP)[16], included cases positive for CK5/6 and/or EGFR, while **BLP 5** included cases positive for CK5/6 and/or EGFR and/or P-cadherin.

Cell culture lysate for proteomics studies

Basal-like breast cancer cell lines MDA-MB-231, MB-468, BT-549, SUM-1315, SUM-159 and Hs 578T and luminal-like breast cancer cell lines MCF7, T47D, HCC1428, SK-Br-3, ZR-73-50 and BT-474 were all obtained from the American Type Culture Collection (ATCC, Manassas, VA). Cell lines were cultured in following medias: MDA-MB-231 in F12 (Sigma-Aldrich) with 10% FBS, 1% Glucose, 1% L-glutamine and 1% P/S. MCF7 and Hs 578T in DMEM (D2650; Sigma-Aldrich) with 10%FBS, 1% L-glutamine, 1% P/S and human recombinant insulin (Sigma-Aldrich). BT-474 in RPMI with 10%FBS, 1%Glucose, 1%L-glutamine and 1% P/S.

Protein extraction, digestion and analysis

Proteins from the cell lysate and the microdissected breast cancer tissue were extracted and enzymatically digested with trypsin, using an in-solution digestion, and the filter-aided samples-preparation (FASP) protocol, respectively, as described in

detail elsewhere[17,18]. After enzymatic digestion of proteins, the resulting peptides were desalted and cleaned using Oasis HLB μElution plates (Waters, Milford, MA, USA). The retrieved peptides were separated by high-pressure liquid chromatography (HPLC) during a biphasic acetonitrile (ACN) gradient and analysed by mass spectrometry: Cell culture lysate on an LTQ-Orbitrap Velos Pro (Thermo Scientific, Bremen, Germany) with a 90 min HPLC gradient; microdissected tumour epithelium from patient tissue on a Q-Exactive HF (Thermo Fisher Scientific, Waltham, MA, USA) with a 180 min gradient.

LC-MS/MS settings used for analysis of microdissected breast cancer tumour cells.

The microdissected samples were analysed in its entirety on a Q-Exactive HF mass spectrometer (Thermo Fisher Scientific, Waltham, MA, USA) connected to a Dionex Ultimate NCR-3500RS LC system. Samples were dissolved in 2% ACN/0.1% formic acid (FA) and trapped on the pre-column (Dionex, Acclaim PepMap 100, 2 cm x 75 µm i.d, 3 µm C18 beads) in loading buffer (0.1% trifluoroacetic acid) at a flowrate of 5 µl/min for 5 minutes, before separation by reverse phase chromatography (PepMap RSLC, 25cm x 75 µm i.d. EASY-spray column, packed with 2µm C18 beads) at a flow of 200 nL/min. Solvent A and B were 0.1% FA (vol/vol) in water and 100% ACN, respectively. The gradient composition was 5% B from 0-5 minutes, which increased linearly to 8 % from 5-5.5 minutes, to 24 % from 5.5-115 minutes, to 35 % B from 115-140 minutes and to 90 % B from 140-155 min. Washing and conditioning of the column were performed from 155-170 minutes with 90 % B, and reduced to 5% B from 170-180 minutes. The MS instrument was equipped with an EASY-spray ion source (Thermo Fisher Scientific, Waltham, MA, USA) and was operated in data-dependent-acquisition mode. Instrument control was performed using Q-Exactive HF Tune 2.4

and Xcalibur 3.0. MS spectra were acquired in the scan range 375 - 1500 m/z with resolution R = 120,000 at m/z 200, with an automatic gain control (AGC) target of 3e6 and a maximum injection time (IT) of 100ms. The 12 most intense eluting peptides above intensity threshold 5E4, with charge states 2 or larger, were sequentially isolated to a target AGC value of 1e5, with resolution R = 30,000, an IT of 110 ms and a normalized collision energy of 28 %. The isolation window was set to 1.6 m/z with an isolation offset of 0.3 and a dynamic exclusion of 25 seconds. Lock-mass internal calibration was used.

Gene expression data analyses

mRNA signatures. A gene expression (mRNA) signature was previously associated with increased microvessel proliferation (proliferative microvessel density; pMVD) in endometrial carcinoma[19]. This signature was associated with aggressive tumour features and reduced survival. Twenty-six of the 32 genes in the vascular proliferation score mapped to the TCGA and METABRIC breast cancer data sets. The resulting 26-gene vascular proliferation scores were generated by subtracting the sum of genes down-regulated in VP-high cases from the sum of genes up-regulated in the same group, as previously described[19]. Also, scores of nestin[11], VEGF[20], hypoxia[21,22], a luminal progenitor signature score and a mature luminal signature score (both scores by Lim et al.)[23], and two scores reflecting proliferation; Oncotype Dx[24] and a PCNA-score[25] were analysed.

From the list of genes differentially expressed between stathmin-high and -low tumours, we identified a stathmin mRNA signature, composed of genes with a fold change ≥2.0 or ≤ -2.0 (FDR<0.006; 332 genes; Supplementary Table S8). By

subtracting the sum of downregulated genes from the sum of upregulated genes in stathmin mRNA-high tumours, a stathmin mRNA signature score was derived for each case.

Connectivity Map. Correlations between the global expression pattern of cases with high stathmin mRNA expression and drug signatures in the Connectivity Map database[26] were explored (TCGA and METABRIC cohorts). Genes differentially expressed (FDR<0.006; fold change ≥2.0 or ≤ -2.0) between tumour subsets of low and high stathmin mRNA levels (cut-point upper quartile) were included in the signature as the basis for the analyses in Connectivity Map.

References

1. Arnes, J.B., *et al.* Expression of epidermal growth factor receptor in relation to BRCA1 status, basal-like markers and prognosis in breast cancer. *J Clin Pathol* **62**, 139-146 (2009).
2. Arnes, J.B., *et al.* Placental cadherin and the basal epithelial phenotype of BRCA1-related breast cancer. *Clin Cancer Res* **11**, 4003-4011 (2005).
3. Arnes, J.B., Collett, K. & Akslen, L.A. Independent prognostic value of the basal-like phenotype of breast cancer and associations with EGFR and candidate stem cell marker BMI-1. *Histopathology* **52**, 370-380 (2008).
4. Arnes, J.B., *et al.* Vascular proliferation is a prognostic factor in breast cancer. *Breast cancer research and treatment* **133**, 501-510 (2012).
5. Collett, K., *et al.* A basal epithelial phenotype is more frequent in interval breast cancers compared with screen detected tumors. *Cancer Epidemiol Biomarkers Prev* **14**, 1108-1112 (2005).
6. Foulkes, W.D., *et al.* The prognostic implication of the basal-like (cyclin E high/p27 low/p53+/glomeruloid-microvascular-proliferation+) phenotype of BRCA1-related breast cancer. *Cancer Res* **64**, 830-835 (2004).
7. Foulkes, W.D., *et al.* Germline BRCA1 mutations and a basal epithelial phenotype in breast cancer. *J Natl Cancer Inst* **95**, 1482-1485 (2003).
8. Goffin, J.R., *et al.* Glomeruloid microvascular proliferation is associated with p53 expression, germline BRCA1 mutations and an adverse outcome following breast cancer. *British journal of cancer* **89**, 1031-1034 (2003).
9. Knutsvik, G., *et al.* Evaluation of Ki67 expression across distinct categories of breast cancer specimens: a population-based study of matched surgical specimens, core needle biopsies and tissue microarrays. *PloS one* **9**, e112121 (2014).
10. Kruger, K., *et al.* Microvessel proliferation by co-expression of endothelial nestin and Ki-67 is associated with a basal-like phenotype and aggressive features in breast cancer. *Breast (Edinburgh, Scotland)* **22**, 282-288 (2013).
11. Kruger, K., *et al.* Expression of Nestin associates with BRCA1 mutations, a basal-like phenotype and aggressive breast cancer. *Scientific reports* **7**, 1089 (2017).
12. Knutsvik, G., Collett, K., Arnes, J., Akslen, L.A. & Stefansson, I.M. QSOX1 expression is associated with aggressive tumor features and reduced survival in breast carcinomas. *Modern pathology : an official journal of the United States and Canadian Academy of Pathology, Inc* **29**, 1485-1491 (2016).
13. Hammond, M.E., Hayes, D.F., Wolff, A.C., Mangu, P.B. & Temin, S. American society of clinical oncology/college of american pathologists guideline recommendations for immunohistochemical testing of estrogen and progesterone receptors in breast cancer. *Journal of oncology practice* **6**, 195-197 (2010).
14. Iwamoto, T., *et al.* Estrogen receptor (ER) mRNA and ER-related gene expression in breast cancers that are 1% to 10% ER-positive by immunohistochemistry. *Journal of clinical oncology : official journal of the American Society of Clinical Oncology* **30**, 729-734 (2012).
15. Fujii, T., *et al.* Revisiting the definition of estrogen receptor positivity in HER2-negative primary breast cancer. *Annals of oncology : official journal of the European Society for Medical Oncology* **28**, 2420-2428 (2017).
16. Tischkowitz, M., *et al.* Use of immunohistochemical markers can refine prognosis in triple negative breast cancer. *BMC cancer* **7**, 134 (2007).

17. Kinter, M. & Sherman, N.E. *Protein sequencing and identification using tandem mass spectrometry*, (John Wiley, New York, 2000).
18. Wisniewski, J.R. Proteomic sample preparation from formalin fixed and paraffin embedded tissue. *Journal of visualized experiments : JoVE* (2013).
19. Stefansson, I.M., *et al.* Increased angiogenesis is associated with a 32-gene expression signature and 6p21 amplification in aggressive endometrial cancer. *Oncotarget* **6**, 10634-10645 (2015).
20. Hu, Z., *et al.* A compact VEGF signature associated with distant metastases and poor outcomes. *BMC Med* **7**, 9 (2009).
21. Eustace, A., *et al.* A 26-gene hypoxia signature predicts benefit from hypoxia-modifying therapy in laryngeal cancer but not bladder cancer. *Clin Cancer Res* **19**, 4879-4888 (2013).
22. Halle, C., *et al.* Hypoxia-induced gene expression in chemoradioresistant cervical cancer revealed by dynamic contrast-enhanced MRI. *Cancer Res* **72**, 5285-5295 (2012).
23. Lim, E., *et al.* Aberrant luminal progenitors as the candidate target population for basal tumor development in BRCA1 mutation carriers. *Nat Med* **15**, 907-913 (2009).
24. Paik, S., *et al.* A multigene assay to predict recurrence of tamoxifen-treated, node-negative breast cancer. *N Engl J Med* **351**, 2817-2826 (2004).
25. Venet, D., Dumont, J.E. & Detours, V. Most random gene expression signatures are significantly associated with breast cancer outcome. *PLoS Comput Biol* **7**, e1002240 (2011).
26. Lamb, J., *et al.* The Connectivity Map: using gene-expression signatures to connect small molecules, genes, and disease. *Science (New York, N.Y.)* **313**, 1929-1935 (2006).
27. Gyorffy, B., *et al.* An online survival analysis tool to rapidly assess the effect of 22,277 genes on breast cancer prognosis using microarray data of 1,809 patients. *Breast cancer research and treatment* **123**, 725-731 (2010).
28. Jiang, H., *et al.* Resveratrol downregulates PI3K/Akt/mTOR signaling pathways in human U251 glioma cells. *J Exp Ther Oncol* **8**, 25-33 (2009).
29. Feng, Z., *et al.* The antipsychotic agent trifluoperazine hydrochloride suppresses triple-negative breast cancer tumor growth and brain metastasis by inducing G0/G1 arrest and apoptosis. *Cell Death Dis* **9**, 1006 (2018).
30. Wang, B., Luo, Y., Zhou, X. & Li, R. Trifluoperazine induces apoptosis through the upregulation of Bax/Bcl2 and downregulated phosphorylation of AKT in mesangial cells and improves renal function in lupus nephritis mice. *Int J Mol Med* **41**, 3278-3286 (2018).
31. Xu, F., *et al.* Repositioning antipsychotic fluphenazine hydrochloride for treating triple negative breast cancer with brain metastases and lung metastases. *Am J Cancer Res* **9**, 459-478 (2019).
32. Liu, Y., Chen, S., Xue, R., Zhao, J. & Di, M. Mefloquine effectively targets gastric cancer cells through phosphatase-dependent inhibition of PI3K/Akt/mTOR signaling pathway. *Biochem Biophys Res Commun* **470**, 350-355 (2016).

Supplementary Figure S1

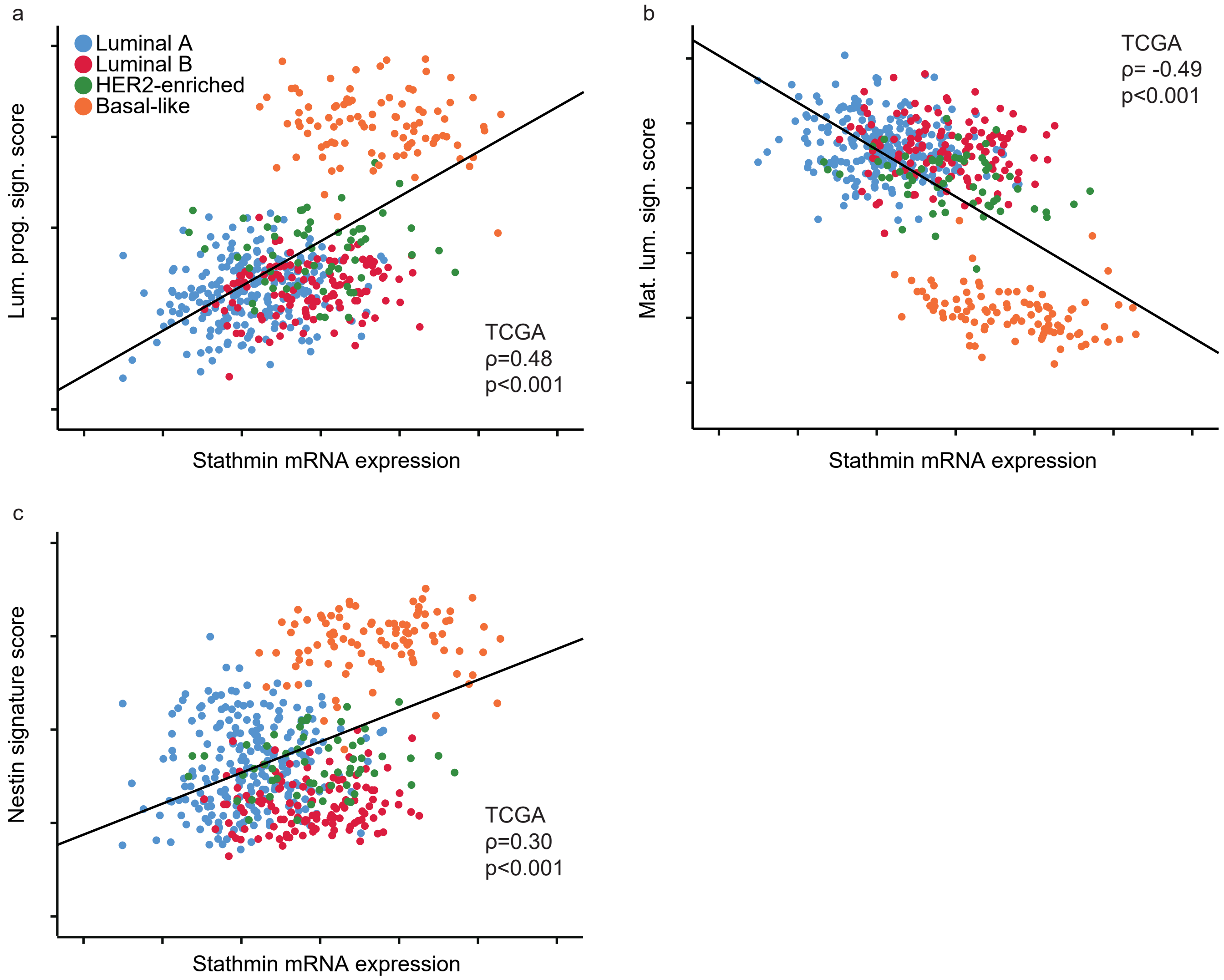

Supplementary Figure S1. Stathmin mRNA expression correlates with stemness. Correlation between stathmin mRNA and a luminal progenitor signature score **(a)**, a mature luminal signature score **(b)** and a nestin signature score **(c)** in the TCGA cohort. The scatter plots are presented with p-values by Spearman's rank correlation and the coefficients (ρ).

Supplementary Figure S2

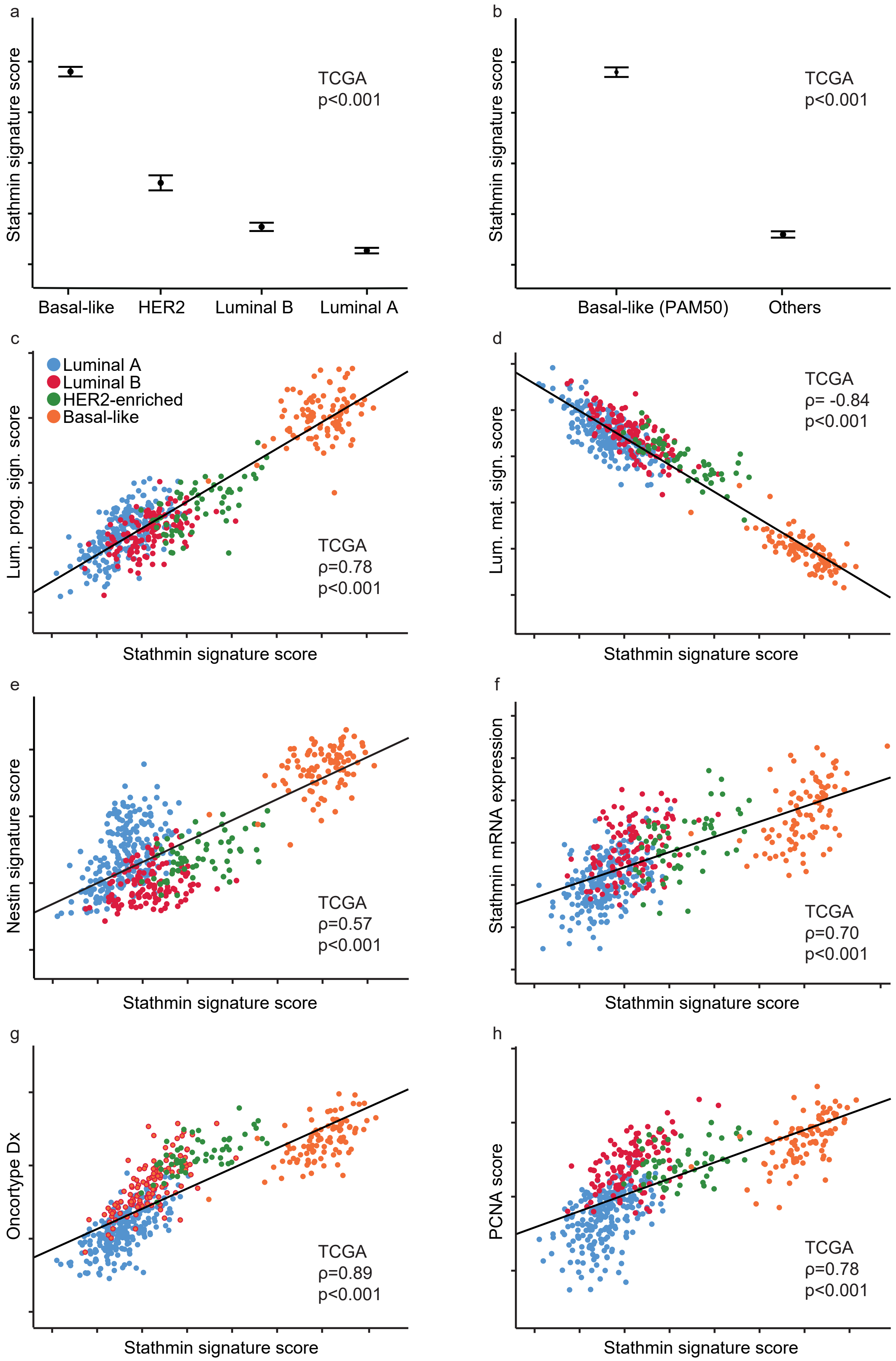

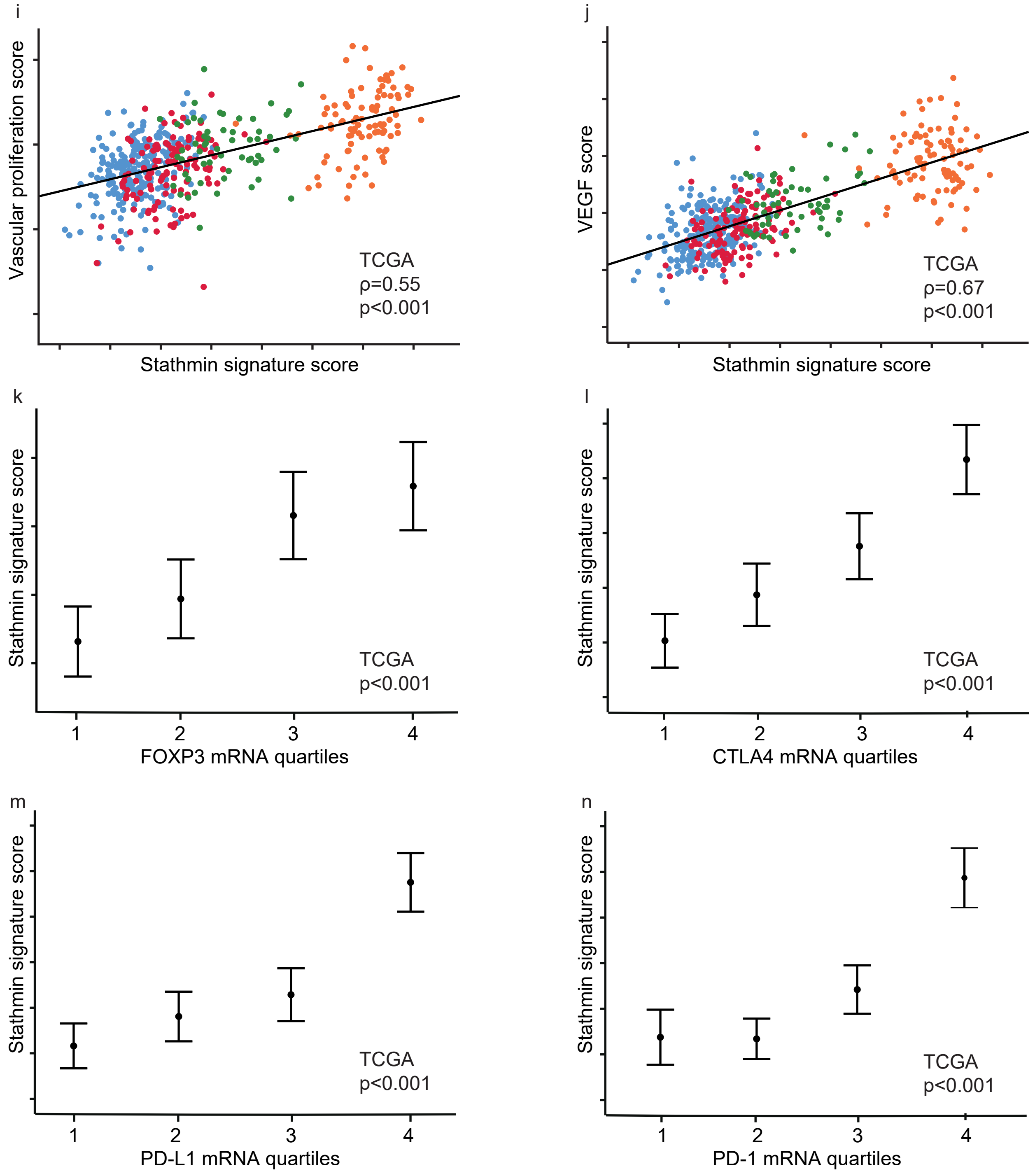
i
Vascular proliferation score
Stathmin signature score
TCGA
ρ=0.55
p<0.001
j
VEGF score
Stathmin signature score
TCGA
ρ=0.67
p<0.001
k
Stathmin signature score
FOXP3 mRNA quartiles
1
2
3
4
TCGA
p<0.001
l
Stathmin signature score
CTLA4 mRNA quartiles
1
2
3
4
TCGA
p<0.001
m
Stathmin signature score
PD-L1 mRNA quartiles
1
2
3
4
TCGA
p<0.001
n
Stathmin signature score
PD-1 mRNA quartiles
1
2
3
4
TCGA
p<0.001

Supplementary Figure S2. The stathmin signature associates with basal-like tumours, features of stemness, proliferation, vascular proliferation and immune-cell activation in the TCGA cohort. Stathmin signature scores across molecular subtypes of breast cancer **(a)** and in basal-like compared to non-basal breast cancer **(b)**. Correlations between stathmin signature score, luminal progenitor **(c)** and mature luminal signature score **(d)**, nestin signature score **(e)**, stathmin mRNA expression **(f)**, Oncotype DX score **(g)**, PCNA score **(h)**, a gene expression vascular score **(i)**, and VEGF score **(j)**. Stathmin signature scores across FOXP3 **(k)**, CTLA4 **(l)**, PD-L1 **(m)** and PD-1 **(n)** mRNA quartiles. Data from the TCGA cohort. Data shown with error-bars representing 95% confidence interval of the mean, and p-values by Kruskal-Wallis test/Mann-Whitney U test. The scatter plots are presented with p-values by Spearman's rank correlation and the coefficients (ρ).

Supplementary Figure S3

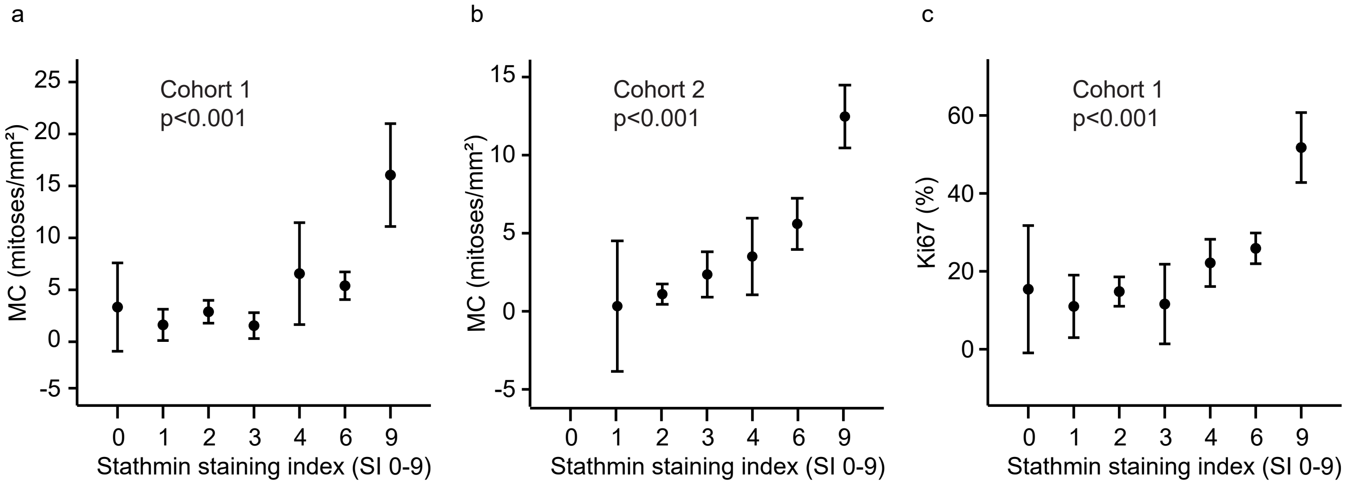

Supplementary Figure S3. Tumour cell proliferation according to stathmin expression levels (staining index 0-9): by mitotic count, MC (mitoses/mm^2) in patient cohort 1 (**a**), cohort 2 (**b**), and by Ki67 (%) in cohort 1 (**c**).

Supplementary Figure S4

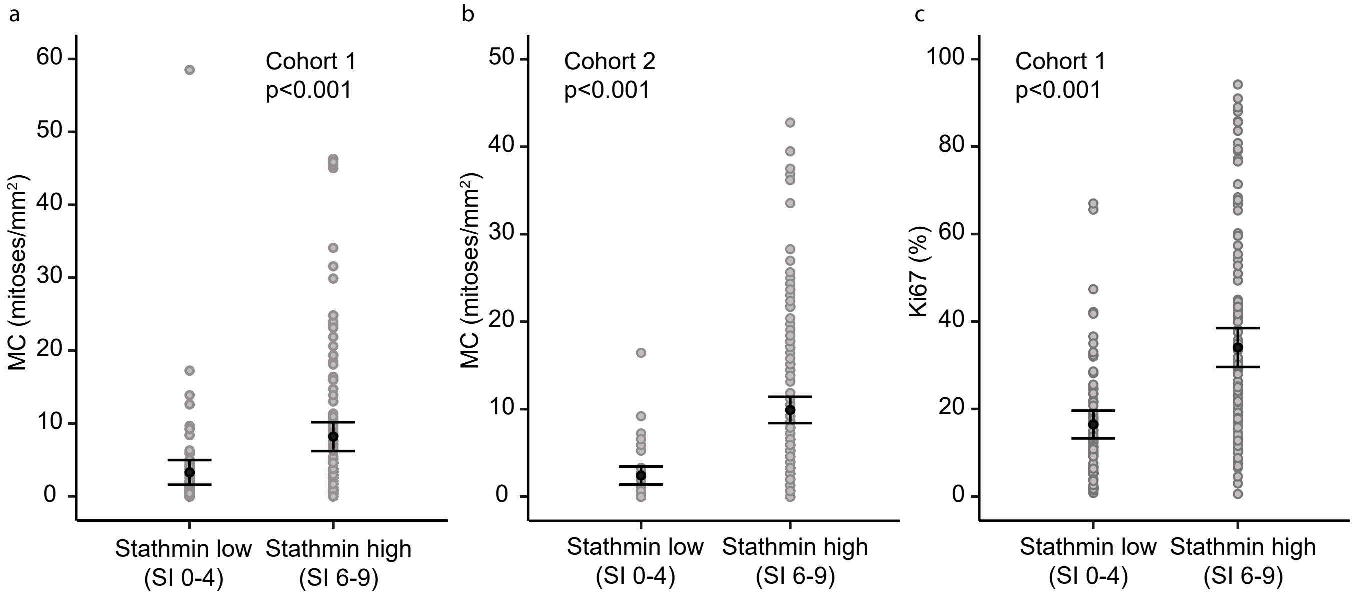

Supplementary Figure S4. **High stathmin associates with proliferation.** Tumour cell proliferation by mitotic count, MC **(a-b)** and Ki67 **(c)** in stathmin low and high groups (by IHC) in breast cancers from the two patient cohorts. Data shown with error-bars on top of scatter plots. Error-bars representing 95% confidence interval of the mean, and p-values by Mann-Whitney U-test.

Supplementary Figure S5

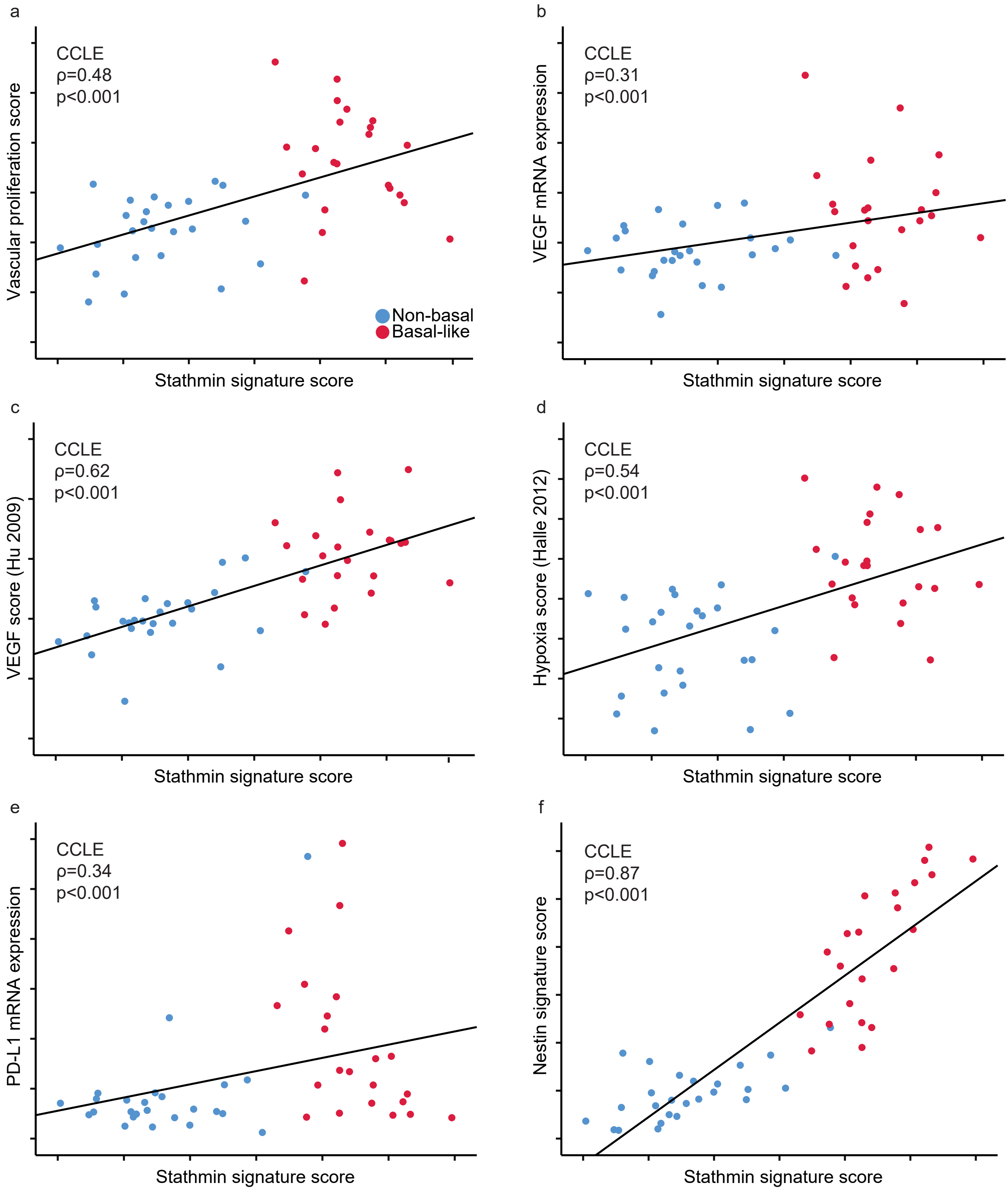

Supplementary Figure S5. The stathmin signature associates with signatures reflecting vascular proliferation, VEGF expression, hypoxia, immune cell activation, and features of stemness in the Cancer Cell Line Encyclopedia. Correlation between stathmin signature score and a gene expression vascular proliferation score (a), VEGF mRNA expression (b), VEGF score (c), hypoxia score (d), PD-L1 mRNA expression (e) and nestin signature score (f); all data are from the breast cancer cell lines of the Cancer Cell Line Encyclopedia. Scatter plots are presented with p-values by Spearman's rank correlation and the coefficients (ρ). Data shown with error-bars representing 95% confidence interval of the mean, and p-values by Kruskal-Wallis test.

Supplementary Figure S6

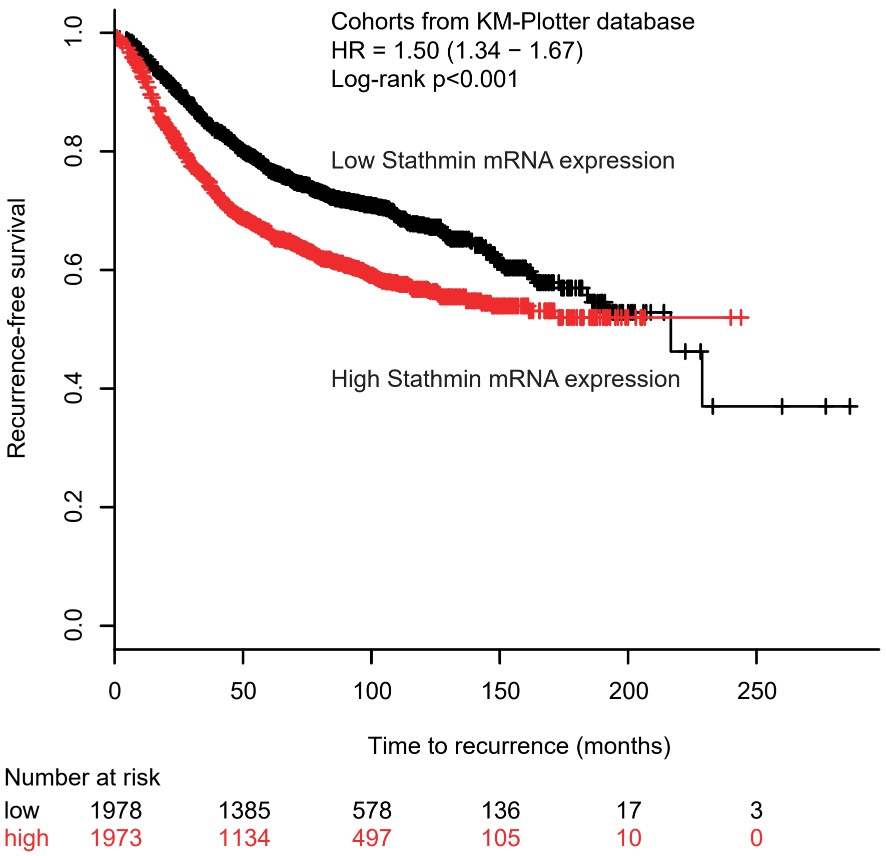

Supplementary Figure S6. Recurrence-free breast cancer survival by stathmin mRNA expression. Kaplan-Meier recurrence-free breast cancer survival according to stathmin mRNA expression in the cohorts from the online "KM plotter" database (www.kmplot.com)[27]. (cut-point by median, log-rank test for difference)**.**

Variables	Stathmin low (n=75) n (%)	Stathmin high (n=112) n (%)	OR	95% CI	P-value[a]
**p53**					0.001
Low, score ≤ 3	70 (45.5)	84 (54.5)	1		
High, score >3	5 (15.2)	28 (84.8)	4.67	1.71, 12.72	
**Nestin[b]**					0.003
Negative, score=0	73 (44.0)	93 (56.0)	1		
Positive, score>0	2 (10.0)	18 (90.0)	7.07	1.59, 31.43	
**pMVD[c]**					0.047
Low (< 4.59)	57 (44.2)	72 (55.8)	1		
High (≥ 4.59)	13 (27.7)	34 (72.3)	2.07	1.00, 4.29	
**VPI[c]**					0.266
Low (< 5.44%)	56 (42.1)	77 (57.9)	1		
High (≥ 5.44%)	14 (32.6)	29 (67.4)	1.51	0.73, 3.11	
**P-cadherin**					0.262
Low, score ≤3	65 (41.9)	90 (58.1)	1		
High, score >3	10 (31.3)	22 (68.8)	1.59	0.71, 3.58	
**EGFR[d]**					0.229
Negative, ≤ 1%	67 (42.7)	90 (57.3)	1		
Positive, > 1%	4 (26.7)	11 (73.3)	2.05	0.63, 6.71	
**TNP**					0.012
Absent	71 (43.6)	92 (56.4)	1		
Present	4 (16.7)	20 (83.3)	3.86	1.26, 11.79	
**BLP 1**					0.004
Absent	74 (43.3)	97 (56.7)	1		
Present	1 (6.3)	15 (93.8)	11.44	1.48, 88.60	
**BLP 2**					0.033
Absent	72 (42.6)	97 (57.4)	1		
Present	3 (16.7)	15 (83.3)	3.71	1.04, 13.30	
**BLP 3[e]**					0.022[f]
Absent	74 (42.3)	101 (57.7)	1		
Present	0 (0.0)	8 (100.0)	-[g]	-	
**BLP 4[e]**					0.001
Absent	73 (44.2)	92 (55.8)	1		
Present	1 (5.3)	18 (94.7)	14.28	1.86, 109.51	
**BLP 5[e]**					0.003
Absent	72 (44.4)	90 (55.6)	1		
Present	3 (12.5)	21 (87.5)	5.60	1.61, 19.52	

Supplementary Table S1. Stathmin protein expression and associations with vascular proliferation- and basal cell markers as well as selected molecular characteristics in breast cancer. Cohort 1 (n=187). n: number of patients; OR: odds ratio; CI: confidence interval; pMVD: proliferative microvessel density; VPI: vascular proliferation index; EGFR: epidermal growth factor receptor; TNP (triple negative phenotype): ER-, PR-, HER2-; BLP (basal-like phenotype) 1: ER-, HER2-, CK5/6+; 2: ER-, HER2-, P-Cadherin+; 3: ER-, HER2-, EGFR+; 4: ER-, HER2-, CK5/6+ and/or EGFR+; 5: ER-, HER2-, CK5/6+ and/or P-cadherin+ and/or EGFR+. [a] Pearson's chi-squared test; [b] One case missing nestin staining; [c] Eleven cases missing information on pMVD (Nestin+/Ki67+ vessels) and VPI (pMVD/MVD); [d] Fifteen cases missing EGFR staining; [e] Four, three and one case(s) missing information on BLP3, BLP4 and BLP5 status; [f] Fisher's exact test; [g] Odds ratio could not be calculated due to zero BLP3 positive cases in the stathmin low group.

Variables	Stathmin low (n=40) n (%)	Stathmin high (n=158) n (%)	OR	95% CI	P-value[a]
**p53[b]**					0.005
Low, score ≤ 3	35 (25.0)	105 (75.0)	1		
High, score >3	4 (7.3)	51 (92.7)	4.25	1.43,12.61	
**Nestin[c]**					<0.001
Neg, score=0	34 (26.2)	96 (73.8)	1		
Pos, score>0	0 (0.0)	51 (100.0)	-[d]	-	
**pMVD[e]**					0.005
Low, < 1.45	36 (24.2)	113 (75.8)	1		
High ≥ 1.45	2 (4.7)	41 (95.3)	6.53	1.51, 28.35	
**VPI[e]**					0.007
< 2.25%	35 (24.3)	109 (75.7)	1		
≥ 2.25%	3 (6.3)	45 (93.8)	4.82	1.41, 16.47	
**P-cadherin[f]**					0.021
Low, score ≤3	28 (25.9)	80 (74.1)	1		
High, score >3	11 (12.6)	76 (87.4)	2.42	1.13, 5.20	
**EGFR[g]**					0.409
Neg, ≤ 1%	33 (20.4)	129 (79.6)	1		
Pos, > 1%	4 (13.8)	25 (86.2)	1.60	0.52, 4.91	
**TNP[h]**					0.006
Absent	33 (25.0)	99 (75.0)	1		
Present	5 (8.1)	57 (91.9)	3.80	1.40, 10.28	
**BLP 1[i]**					0.001
Absent	40 (24.8)	121 (75.2)	1		
Present	0 (0.0)	35 (100.0)	-[j]	-	
**BLP 2[i]**					0.004
Absent	34 (25.6)	99 (74.4)	1		
Present	5 (8.1)	57 (91.9)	3.92	1.45, 10.58	
**BLP 3[i]**					0.023[k]
Absent	38 (22.0)	135 (78.0)	1		
Present	0 (0.0)	19 (100.0)	-[j]	-	
**BLP 4[i]**					0.001
Absent	38 (24.8)	115 (75.2)	1		
Present	0 (0.0)	39 (100.0)	-[j]	-	
**BLP 5[i]**					0.003
Absent	34 (26.4)	95 (73.6)	1		
Present	5 (7.8)	59 (92.2)	4.22	1.56, 11.40	

Supplementary Table S2. Stathmin protein expression and associations with vascular proliferation and basal cell markers as well as selected molecular characteristics in breast cancer. Cohort 2 (n=198). n: number of patients; OR: odds ratio; CI: confidence interval; pMVD: proliferative microvessel density; VPI: vascular proliferation index; EGFR: epidermal growth factor receptor; TNP (triple negative phenotype): ER-, PR-, HER2-; BLP (basal-like phenotype) 1: ER-, HER2-, CK5/6+; 2: ER-, HER2-, P-Cadherin+; 3: ER-, HER2-, EGFR+; 4: ER-, HER2-, CK5/6+ and/or EGFR+; 5: ER-, HER2-, CK5/6+ and/or P-cadherin+ and/or EGFR+. [a] Pearson Chi-square; [b] Three cases missing p53 staining; [c] Seventeen cases missing nestin staining; [d] Odds ratio could not be calculated due to zero nestin positive cases in the stathmin low group; [e] Six cases missing information on pMVD (Factor VIII/Ki67) and VPI (pMVD/MVD); [f] Three cases missing P-cadherin staining; [g] Seven cases missing EGFR staining; [h] Four cases missing information on TNP status; [i] Two, three, six, six and five cases missing information on BLP 1, 2, 3, 4 and 5 status; [j] Odds ratio could not be calculated due to zero BLP 1, 3 and 4 positive cases in the stathmin low group; [k] Fisher`s exact test.

	METABRIC discovery cohort (n=939)[a]					METABRIC validation cohort (n=845)[a]				
	Stathmin mRNA low n (%)	Stathmin mRNA high n (%)	OR	95% CI	P-value[b]	Stathmin mRNA low n (%)	Stathmin mRNA high n (%)	OR	95% CI	P-value[b]
**Tumour diameter**					0.093					0.019
≤ 20 mm	214 (52.3)	195 (47.7)	1			190 (54.8)	157 (45.2)	1		
> 20 mm	248 (46.8)	282 (53.1)	1.24	0.96, 1.61		232 (46.6)	266 (53.4)	1.39	1.05, 1.83	
**Histologic grade[c]**					<0.001					<0.001
Grade 1-2	302 (66.2)	154 (33.8)	1			238 (65.6)	125 (34.4)	1		
Grade 3	160 (33.1)	323 (66.9)	3.96	3.01, 5.19		137 (33.5)	272 (66.5)	3.78	2.81, 5.10	
**Nodal status**					0.052					0.653
Negative	252 (52.3)	230 (47.7)	1			223 (50.7)	217 (49.3)	1		
Positive	210 (46.0)	247 (54.0)	1.29	0.997, 1.67		199 (49.1)	206 (50.9)	1.06	0.81, 1.39	
**ER[d]**					<0.001					<0.001
Positive	435 (57.5)	321 (42.5)	1			361 (61.6)	225 (38.4)	1		
Negative	27 (14.8)	156 (85.2)	7.83	5.08, 12.08		43 (19.3)	180 (80.7)	6.71	4.63, 9.74	
**Mol. subtypes**					<0.001					<0.001
Luminal A	351 (75.3)	115 (24.7)				223 (87.5)	32 (12.5)			
Luminal B	86 (32.1)	182 (67.9)				112 (50.0)	112 (50.0)			
HER2 enriched	19 (21.8)	68 (78.2)				62 (40.5)	91 (59.5)			
Basal-like	6 (5.1)	112 (94.9)				25 (11.7)	188 (88.3)			
**Mol. subtypes**					<0.001					<0.001
Non-basal	456 (55.5)	365 (44.5)	1			397 (62.8)	235 (37.2)	1		
Basal-like	6 (5.1)	112 (94.9)	23.32	10.14, 53.62		25 (11.7)	188 (88.3)	12.7	8.12, 19.87	

Supplementary Table S3. Stathmin mRNA expression and associations with clinico-pathological characteristics and molecular subtypes of breast cancer in the METABRIC discovery (n=939) and validation cohorts (845). [a] The normal breast-like category is excluded; [b] Pearson's chi-squared test; [c] Seventy-three cases missing information on histologic grade in the validation cohort; [d] Thirty-six cases missing information on ER status in the validation cohort.

	TCGA breast cancer cohort (n=505)[a]				
	Stathmin mRNA low n (%)	Stathmin mRNA high n (%)	OR	95% CI	P-value[b]
**ER[c]**					<0.001
Positive	328 (85.4)	56 (14.6)	1		
Negative	45 (39.5)	69 (60.5)	8.98	5.61, 14.38	
**Molecular subtypes**					<0.001
Luminal A	185 (80.4)	45 (19.6)			
Luminal B	45 (36.9)	77 (63.1)			
HER2 enriched	17 (29.3)	41 (70.7)			
Basal-like	5 (5.3)	90 (94.7)			
**Molecular subtypes**					<0.001
Non-basal	348 (84.9)	62 (15.1)	1		
Basal-like	31 (32.6)	64 (67.4)	11.59	6.98, 19.23	
**BRCA status[d]**					0.021
BRCA1/2 wild type	347 (76.3)	108 (23.7)	1		
BRCA1 mutated	6 (46.2)	7 (53.8)	3.74	1.23, 11.39	

Supplementary Table S4. Stathmin mRNA expression and associations with ER status, molecular subtypes of breast cancer and the BRCA1 genotype in the TCGA cohort (n=505). [a] The normal breast-like category is excluded; [b] Pearson's chi-squared test; [c] Seven cases missing information on ER status; [d] Thirty-seven cases missing information on BRCA status.

Cohort 1	Unadjusted model				Adjusted model, n=183		
**Variables**	**n**	**OR**	**95% CI**	**P-value**	**OR**	**95% CI**	**P-value**
**Histologic grade**				<0.001			0.214
Grade 1-2	147	1			1		
Grade 3	37	9.60	3.45, 26.75		2.26	0.62, 8.23	
**Ki67**				<0.001			0.003
≤31.5%	126	1			1		
>31.5%	57	57.67	7.46, 446.14		15.20	1.70, 136.18	
**p53**				<0.001			0.002
Low, score ≤ 3	152	1			1		
High, score > 3	32	22.87	7.37, 70.95		7.01	1.93, 25.45	
**Stathmin**				<0.001			0.161
Low, score ≤ 4	74	1			1		
High, score > 4	110	14.28	1.86, 109.5		4.01	0.44, 36.12	

Supplementary Table S5. Prediction of the core basal phenotype (ER-, HER2-, CK5/6+ and/or EGFR+) by logistic regression. Cohort 1 (n=187). n: number of cases; OR: odds ratio; CI: confidence interval.

Cohort 1	Unadjusted model				Adjusted model, n=186		
**Variables**	**n**	**OR**	**95% CI**	**P-value**	**OR**	**95% CI**	**P-value**
**Histologic grade**				<0.001			0.001
Grade 1-2	148	1			1		
Grade 3	38	9.66	4.31, 21.65		4.99	1.95, 12.82	
**ER**				<0.001			0.044
Pos, ≥10%	144	1			1		
Neg, <10%	42	7.93	3.71, 16.96		2.77	1.04, 7.38	
**HER2[a]**				0.003			0.017
Neg	159	1			1		
Pos	27	3.48	1.51, 8.04		3.42	1.25, 9.36	
**p53**				<0.001			0.012
Low, score ≤3	154	1			1		
High, score >3	32	11.00	4.53, 26.73		4.10	1.36, 12.32	
**CK5/6**				<0.001			0.157
Neg, score=0	157	1			1		
Pos, score>0	29	5.95	2.55, 13.90		2.27	0.74, 6.96	
**Stathmin**				<0.001			0.031
Low, score ≤4	75	1			1		
High, score >4	111	4.12	1.96, 8.66		2.55	1.07, 6.08	

Supplementary Table S6. Prediction of proliferation (Ki67) by logistic regression. Cohort 1 (n=187). n: number of cases; OR: odds ratio; CI: confidence interval; [a] HER2 positive cases: HER2 IHC 3+ and HER2 IHC 2+ with a HER2/Chr17 ratio by SISH ≥ 2.0.

Rank	CMAP compound	n	Enrichment score	P-value	Comment
1	Resveratrol	9	-0.845	0	Proposed PI3K inhibition[28]
2	Trifluoperazine	16	-0.663	0	Proposed PI3K inhibition[29,30]
3	Fluphenazine	18	-0.567	0.00002	Proposed PI3K inhibitory mechanism[31]
4	0175029-0000	6	-0.801	0.00016	
5	1,4-chrysenequinone	2	-0.983	0.00066	
6	Tretinoin	22	-0.411	0.00072	All-trans retinoic acid
7	0173570-0000	6	-0.728	0.00077	
8	Mefloquine	5	-0.779	0.00094	Proposed PI3K inhibitory mechanism[32]
9	Astemizole	5	-0.771	0.00118	Anti-histamine
10	Wortmannin	18	-0.437	0.00151	PI3K inhibitor

Supplementary Table S7. Connectivity Map (CMAP) analysis; list of compounds with a possible potential to drive stathmin-high tumours into a stathmin-low state. The expression changes from the compounds tested were scored according to the stathmin signature score levels. The P-value for each compound represents the distribution of this score in the n instances, compared with the distribution of these scores among all compounds tested, using a permutation test[26].

Gene Symbol				
ABAT	CHEK1	GJA1	MUC1	SEC14L4
ABCC11	CHODL	GLYATL2	MUM1L1	SEMA3C
ABCC8	CHRNA5	GOLSYN	MYBL2	SERPINA11
ACADSB	CHST8	GP2	NAT1	SERPINA3
ACE2	CLGN	GPSM2	NAT2	SERPINA5
ACMSD	CLIC6	GREB1	NCAM2	SERPINA6
ACOX2	CLSTN2	GRP	NDC80	SERPINB5
ACSM1	CMBL	GRPR	NEIL3	SFRP1
AGR2	CNTD1	GSTM3	NEK10	SH3GL3
AGR3	COCH	HIST1H1A	NME5	SHC4
AGTR1	CPA3	HORMAD1	NMU	SIDT1
ANKRD30A	CPB1	HPDL	NOL4	SLC16A6
ANKRD43	CRABP1	HPN	NPNT	SLC26A9
ANLN	CRIP1	HPX	NPY1R	SLC39A6
ANXA9	CST9	HRASLS	NTN4	SLC40A1
AR	CST9L	HRASLS3	NUF2	SLC44A4
ARHGAP11A	CTSL2	IGFALS	OCA2	SLC6A14
ARL9	CXCL1	IL12RB2	OGN	SLC7A2
ART3	CYP21A2	IL20RA	OMD	SLC7A8
ASPM	CYP39A1	IL22RA2	ORC1L	SLITRK6
ATP1A2	CYP4B1	IL6ST	ORC6L	SMC1B
ATP6V1C2	CYP4X1	INDO	OVOS2	SOSTDC1
B3GNT5	CYP4Z1	INPP4B	OXGR1	SOX11
BCAS1	DACH1	KCNE4	PCDH8	SPATA4
BCL11A	DCC1	KCNJ3	PDZK1	SPC25
BLM	DCDC2	KCNK5	PGLYRP2	SPDEF
BUB1	DDC	KIAA1370	PGR	SPINK4
C10orf82	DEPDC1	KIF14	PHGDH	SRrp35
C15orf42	DEPDC1B	KIF15	PHYHD1	STAC
C16orf45	DIO1	KIF18A	PIP	STC2
C18orf56	DKFZp762E1312	KIF1A	PKIB	STIL
C19orf21	DLG7	KIF2C	PKP1	STMN1
C1orf135	DNAJC12	KLK6	PLAT	SUSD3
C1orf34	DNALI1	KLK8	POTE15	SYT17
C1orf64	DSC2	KRT16	POU4F1	TBC1D9
C20orf103	DSC3	KRT222P	PPP1R14C	TFAP2B
C20orf114	DYNLRB2	KRT37	PPP1R3C	TFF1
C20orf39	EGFR	LAMP3	PRDM13	TFF3
C4orf18	ELF5	LCT	PREX1	THBS4
C6orf173	EN1	LDLRAD1	PROM1	THSD4
C6orf97	ERBB4	LEMD1	PRR15	TMC5
C8orf47	ESR1	LHX2	PSAT1	TMEM26
C9orf58	EXO1	LMO4	PTCHD1	TMEM45B
CA12	FABP7	LOC124220	PTPRT	TMSL8
CACNG1	FAM54A	LOC130576	PTX3	TPX2
CALML5	FAM64A	LONRF2	RAD51AP1	TRH
CAPN13	FAM77C	LRG1	RAI2	TSPAN1

CAPN9	FAM79B	LRP8	RARRES1	TTK
CASC1	FAM81B	LRRC17	RDHE2	TYMS
CCDC48	FAM83D	LRRC48	REEP6	UBE2C
CCKBR	FANCA	LY6D	RERG	UGT2B11
CCNA2	FBP1	MAPK4	RET	UGT2B15
CCNB2	FBXL16	MATN3	RGS22	UGT8
CCNE1	FGFBP1	MATN4	RLBP1	VGLL1
CCNE2	FLJ45557	MCM10	RNF182	WISP3
CDC20	FLRT3	MELK	ROPN1	WNK4
CDC45L	FMO5	METRN	ROPN1B	XBP1
CDC7	FOXA1	MEX3A	ROPN1L	XK
CDCA5	FOXC1	MGC10981	RRM2	ZBTB16
CDCA7	FOXM1	MIA	S100A8	ZIC1
CDCA8	FSIP1	MICALCL	SAG	ZMYND10
CDKN2A	FZD9	MKI67	SCGB1D1	ZNF552
CEACAM6	GABRP	MLPH	SCGB1D2	ZNF695
CENPA	GAMT	MMP1	SCGB2A2	ZNF711
CENPF	GATA3	MMP12	SCNN1A	
CEP55	GFRA1	MND1	SCRG1	
CFB	GGH	MS4A2	SCUBE2	

Supplementary Table S8: Genes included in the stathmin mRNA signature.

Genes differentially expressed between stathmin -high and -low cases (sorted alphabetically).
